# A minimal-data approach for spatially resolved parameter analysis of coupled graphene nanomechanical resonators

**DOI:** 10.1126/sciadv.adq0621

**Published:** 2025-11-14

**Authors:** Brittany Carter, Viva R. Horowitz, Uriel Hernandez, David J. Miller, Andrew Blaikie, Benjamín J. Alemán

**Affiliations:** ^1^ Department of Physics, University of Oregon, Eugene, OR 97403, USA.; ^2^ Materials Science Institute, University of Oregon, Eugene, OR 97403, USA.; ^3^ Center for Optical, Molecular, and Quantum Science, University of Oregon, Eugene, OR 97403, USA.; ^4^ Physics Department, Hamilton College, Clinton, NY 13323, USA.; ^5^ Phil and Penny Knight Campus for Accelerating Scientific Impact, University of Oregon, Eugene, OR 97403, USA.

## Abstract

Networks of nanoelectromechanical (NEMS) resonators are useful analogs for a variety of many-body systems and enable applications in sensing, phononics, and mechanical information processing. A challenge toward realizing practical NEMS networks is the ability to characterize the constituent resonator building blocks and their coupling. Here, we spatially map graphene NEMS networks and introduce an efficient algebraic formalism to quantify the site-specific elasticity, mass, damping, and coupling parameters of network clusters. In a departure from multiple regression, our algebraic analysis uses minimal measurements to fully characterize the network parameters without a priori parameter estimates or iterative computation. We apply this suite of techniques to single-resonator and coupled-pair clusters and find excellent agreement with expected parameter values and broader spectral response. Our approach provides a nonregressive framework for accurately characterizing a range of classical and quantum resonator systems, offering a versatile modeling tool applicable across multiple disciplines and advancing the development of programmable NEMS networks.

## Teaser

Efficiently imaging and analyzing the properties of tiny mechanical networks for cutting-edge technology.

## INTRODUCTION

Interacting many-body systems are abundant in nature at a variety of length scales, from atomic solids to the human brain and even celestial bodies. These assemblies also comprise a growing number of diverse synthetic systems, such as solid-state and optical qubit arrays (*1*–*3*), photonic/phononic crystals (*4*, *5*), and neural networks (*6*), and are central to many hot-topic applications ranging from neuromorphic (*7*) and quantum computing (*2*) to strongly correlated phases (*3*) and metamaterials (*8*). Consequently, there is a vibrant effort to understand, control, and engineer the collective behavior of these complex systems.

A compelling experimental analog for these assemblies is the programmable network of nanoelectromechanical (NEMS) resonators (*9*, *10*). In addition to serving as a testbed, such networks promise powerful applications in reconfigurable phononic crystals and waveguides (*11*, *12*), tunable thermal transport (*10*, *13*), and mechanics-based computation and simulation (*9*, *14*, *15*). Recent advancements include demonstrations of collective phenomena in small-scale assemblies (*9*, *10*, *15*), methods for site-specific tuning of individual resonators (*16*), and control over pairwise coupling (*17*–*19*).

However, a critical barrier remains: the lack of scalable, site-specific tools for extracting the physical parameters of individual resonators, and their couplings from network measurements. Standard techniques, while rooted in well-established regression and fitting, are increasingly impractical in the many-body regime due to a combination of inefficiency, inaccuracy, and the need for a priori knowledge (*20*). For example, resonance spectra become crowded and ambiguous as networks grow, making it difficult to identify contributions from specific resonators or weak interactions. Hybridized modes cannot be unambiguously mapped to specific locations, and weak couplings, often vital for emergent behavior (*15*, *21*, *22*), may vanish below noise thresholds (*23*). Furthermore, conventional parameter extraction methods like nonlinear least squares often require initial guesses, extensive data, and iterative fitting, making them susceptible to local minima and high uncertainty (*20*).

Here, we introduce a framework, NetMAP (network mapping and analysis of parameters), which extracts site-specific mechanical properties of coupled nanomechanical networks directly from sparse measurements, with no a priori knowledge, and in a computationally nimble manner. NetMAP solves an inverse algebraic problem in reciprocal space to determine elasticity, mass, damping, and coupling for each node in the network, using just two spatially resolved response vectors (*10*, *24*–*28*). Crucially, it does so without model fitting, without training data, and without initial parameter guesses.

This approach challenges the prevailing assumption that accurate parameter inference requires either dense data, extensive computation, or predefined model constraints. Instead, NetMAP enables efficient, direct, and interpretable extraction of mechanical parameters from complex networks using minimal input. While we implement and validate NetMAP on graphene membrane resonator arrays, the method is domain-agnostic and directly generalizable to any system governed by coupled linear differential equations, including photonic, electronic, or even biological networks. In combination with existing tools for tuning resonators (*16*), NetMAP provides a critical missing capability for programmable nanomechanical networks and may find wide relevance in fields that rely on efficient inverse modeling from sparse or ambiguous data.

## RESULTS

**Fig. 1.**
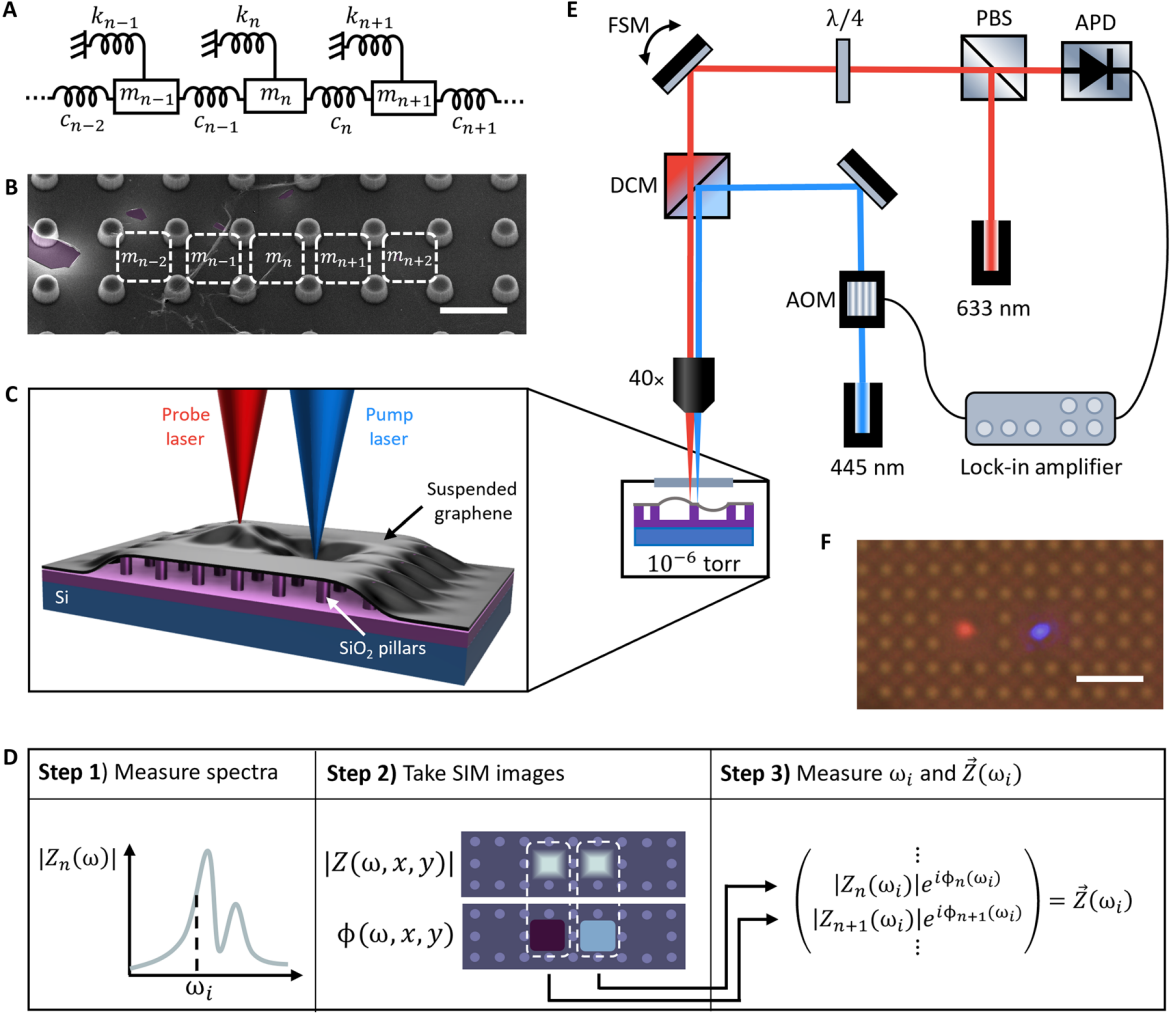
Suspended graphene platform and measurement scheme. (**A**) Linear mass and spring model showing intrinsic springs $\left(k_{n}\right)$, coupling springs $\left(c_{n}\right)$, and masses $\left(m_{n}\right)$. (**B**) SEM image of graphene suspended over pillars. Suspended regions between pillars are depicted as a linear chain of masses. Scale bar, 3μm. (**C**) Cross-sectional view of suspended graphene device showing Si base with SiO_2_ pillars and suspended graphene. The depicted pump and probe lasers are aligned to drive the right-side resonator and measure the motion of the left-side resonator. (**D**) Steps for measuring $\vec{Z}\left(\omega_{i}\right)$ showing (1) an amplitude spectrum of the *n*th resonator, (2) spatial images of a cluster of two resonators, and (3) the complex response vector of the cluster when driven at $\omega_{i}$. (**E**) Schematic of optical set up showing 445-nm pump laser modulated with an AOM and coupled into the optical path with a dichroic mirror (DCM). The 633-nm probe laser is deflected by a polarizing beam splitter (PBS) and polarized by a quarter wave plate (λ/4). The probe position is controlled with a fast-scanning mirror (FSM). The reflected probe passes back through the PBS, and the interference signal is detected by an avalanche photodiode (APD) and resolved by a lock-in amplifier. Both the pump and probe lasers are focused onto the sample through a 40× objective lens, and the sample is under vacuum at $10^{-6}\;$ torr. (**F**) Top-down optical image of the sample under vacuum, with pillars seen as small orange dots. Here, the pump (blue) and probe (red) beams are focused onto neighboring resonator regions of suspended graphene, with a scale bar of 6μm.

In our approach, we model the resonator network as a linear chain of masses and springs, depicted in Fig. 1A. The system of equations describing this model can be organized into matrix form

$$M\ddot{\vec{x}}+B\dot{\vec{x}}+K\vec{x}=\vec{F}e^{i\omega t}$$ (1)

where M, B, and K, are the modal mass, damping, and elasticity matrices, respectively. Using $x_{n}\left(t\right)=|Z_{n}\left(\omega \right)|e^{i\left[\omega t-\phi_{n}\left(\omega \right)\right]}\;$ as the response of the *n*th resonator, we can write Eq. 1 in steady-state form

$$M\left(\omega \right)\vec{Z}\left(\omega \right)=\vec{F}$$ (2)

Here, ω is the drive frequency of $\vec{F}$, $M\left(\omega \right)\equiv -\omega^{2}M+i\omega B+K$, and $\vec{Z}\left(\omega \right)$ is a vector of the complex responses of each resonator in the network, $Z_{n}\left(\omega \right)$. Our analysis approach determines M, B, and K by measuring the response vector $\vec{Z}\left(\omega \right).$ For finite clusters of size N, the edge coupling spring constants vanish ($c_{n-1}\sim 0$ and $c_{n+N+1}\sim 0$) so that M(ω) has 4N unknown parameters. For each measurement of $\vec{Z}\left(\omega \right)$, Eq. 2 provides 2N equations, so to determine the unknown parameters of M, B, and K, $\vec{Z}\left(\omega \right)$ must be measured at a minimum of two drive frequencies, $\omega_{a}$ and $\omega_{b}$ [see the Supplementary Materials and (*29*)]. We combine and reorganize the equations corresponding to $\vec{Z}\left(\omega_{a}\right)$ and $\vec{Z}\left(\omega_{b}\right)$ in Eq. 2 to obtain

$$Z\vec{p}=\vec{0}$$ (3)

where Z is a 4N×4N real-valued matrix of known coefficients determined by $\omega_{a}$, $\omega_{b}$, $\vec{Z}\left(\omega_{a}\right)$, and $\vec{Z}\left(\omega_{b}\right)$. The parameters vector, $\vec{p}$, is a 4N-dimensional vector comprising all the unknown elements of M, B, K, and $\vec{F}$.

To solve Eq. 3 for $\vec{p},$ we resolve the null space of Z via singular value decomposition (SVD). To do so, we use the NumPy package SVD function in Python, which outputs normalized values of $\vec{p}$. The parameters vector $\vec{p}$ can be determined uniquely by separately determining one parameter; otherwise, $\vec{p}$ is unique only up to a multiplicative constant. Regardless, $\vec{p}\;$ can be used to calculate any ratio of interest (e.g., the quality factor and coupling strength) or to compute any function of the model parameters in $\vec{p}\;$ given each term in the function is formulated so that the arbitrary multiplicative constant from the general solution cancels out; as an important example function, the predicted $\vec{Z}\left(\omega \right)$ can be formulated uniquely to validate against the full measured spectra.

We demonstrate NetMAP on network clusters of graphene resonators suspended over Si/SiO_2_ pillar arrays (see Materials and Methods). A scanning electron microscopy (SEM) image of a completed array is shown as one possible coupled configuration in Fig. 1B. We measure the amplitude and phase of the suspended graphene resonators using an optical pump/probe method to thermally drive the resonator and readout the corresponding motion, as shown in Fig. 1 (C, E, and F) (see Materials and Methods). We first apply NetMAP to single resonators, the most familiar case in the micro/nanoelectromechanical systems (MEMS/NEMS) community, to validate its accuracy and provide a more efficient alternative to traditional nonlinear regression. We then extend our analysis to coupled resonators, where the increased system complexity provides a strong benchmark for our approach.

With the goal of determining $\vec{p}\;$ for a local cluster of suspended graphene resonators, we must first construct the matrix Z, which we achieve in three steps (Fig. 1D). First, we measure amplitude and phase spectra and determine a range of drive frequencies ω that provide large signal-to-noise ratios (SNRs) for $|Z_{n}\left(\omega \right)|$. Second, we quantify the size and spatial configuration of the cluster from images of the mechanical motion obtained with scanning interferometric microscopy (SIM) (*24*). Last, we determine $\vec{Z}\left(\omega_{a}\right)$ and $\vec{Z}\left(\omega_{b}\right)$ by using a phase-locked loop (PLL). After completing the PLL measurements, we use the values of $\omega_{a},$ $\omega_{b}$, $\vec{Z}\left(\omega_{a}\right)$, and $\vec{Z}\left(\omega_{b}\right)$ to construct Z (see Materials and Methods).

**Fig. 2.**
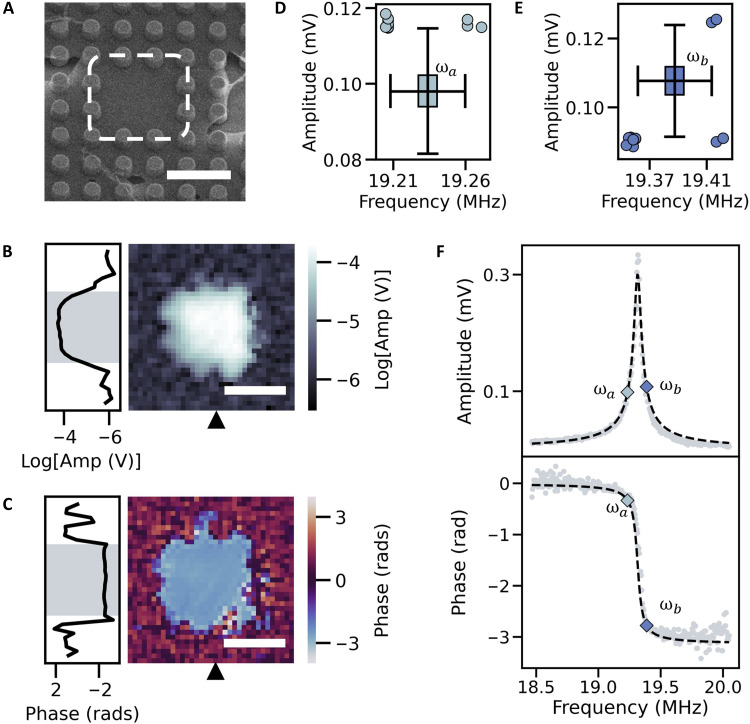
NetMAP analysis of N=1 cluster. (**A**) SEM image of driven uncoupled resonator, with pillar radii of 0.5μm and pillar pitch 2μm. Scale bar, 4μm. (**B**) Amplitude (Amp) and (**C**) phase spatial maps at a drive frequency of 18.81MHz. Scale bars, 5μm. Phase spatial map shows uncorrected wrapped phase values. Black triangles on the bottom axis indicate the location of the vertical line scan on the left-side axis. 2D boxplot of PLL measurement distribution of frequency and amplitude for (**D**) phase lock of $\phi \left(\omega_{a}\right)=1.84\;\text{rad}$ and (**E**) phase lock of $\phi \left(\omega_{b}\right)=-1.28\;\text{rad}$. 2D boxplots shows median, upper, and lower quartile ranges with whiskers that extend to include 1.5 interquartile range. Plotted circles represent data points that were outliers in both frequency and amplitude. (**F**) Amplitude and corrected phase spectra of driven resonator. The diamond points in the amplitude spectrum (top) correspond to the mean PLL measurements of amplitude [$\bar{A}\left(\omega_{a}\right)=0.09827\pm 0.00002$ mV and $\bar{A}\left(\omega_{b}\right)=0.10758\pm 0.00002$ mV] and frequency [$\bar{\Omega }\left(\omega_{a}\right)\;/\;2\pi =19.2337\pm 0.00003\;\text{MHz}$ and $\bar{\Omega }\left(\omega_{b}\right)\;/\;2\pi =19.3876\pm 0.00004\;\text{MHz}$], where the uncertainties are SE. The diamond points in the phase spectrum (bottom) correspond to the locked phase values, $\phi \left(\omega_{a}\right)$ and $\phi \left(\omega_{b}\right)$, and the mean frequency values, $\bar{\Omega }\left(\omega_{a}\right)\;/\;2\pi$ and $\bar{\Omega }\left(\omega_{b}\right)\;/\;2\pi$. The black dotted line represents ∣Z(ω)∣ and ϕ(ω) generated from the normalized $\vec{p}$.

As a first application of NetMAP, we determined the mechanical parameters of the simplest network cluster of size N=1, corresponding to a single, uncoupled resonator. The single resonator device is highlighted in the SEM shown in Fig. 2A, with corresponding SIM images of the amplitude and phase in Fig. 2 (B and C) (see the Supplementary Materials). To compute Z, we chose $\omega_{a}$ and $\omega_{b}$ to be on each side of the resonance peak, shown in Fig. 2F (top). We then used the corrected phase spectra (see the Supplementary Materials), shown in Fig. 2F (bottom), to map the chosen values of $\omega_{a}$ and $\omega_{b}$ to phase values for the PLL. The measured PLL time series of amplitude, $A\left(\omega_{a,b}\right)$, and frequency, $\Omega \left(\omega_{a,b}\right)/2\pi$, are shown as two-dimensional (2D) boxplots for phase lock $\phi \left(\omega_{a}\right)$ in Fig. 2D and for $\phi \left(\omega_{b}\right)$ in Fig. 2E. Using the mean values of amplitude, $\bar{A}\left(\omega_{a}\right)$ and $\bar{A}\left(\omega_{b}\right)$, and frequency, $\bar{\Omega }\left(\omega_{a}\right)$ and $\bar{\Omega }\left(\omega_{b}\right)$, we obtain $\omega_{a}=\bar{\Omega }\left(\omega_{a}\right)$, $\omega_{b}=\bar{\Omega }\left(\omega_{b}\right),$ $\vec{Z}\left(\omega_{a}\right)=\left\{\bar{A}\left(\omega_{a}\right)e^{i\phi \left(\omega_{a}\right)}\right\}$, and $\vec{Z}\left(\omega_{b}\right)$ $=\left\{\bar{A}\left(\omega_{b}\right)e^{i\phi \left(\omega_{b}\right)}\right\}$.

To solve for the parameters vector, $\vec{p}$, we populate Z with coefficients of the experimentally measured $\omega_{a},\omega_{b},\vec{Z}\left(\omega_{a}\right)$ and $\vec{Z}\left(\omega_{b}\right)$. For a single-resonator cluster, the reduced system of equations $Z\vec{p}=\vec{0}$ is

$$\begin{array}{c} \begin{array}{c} \left(\begin{array}{cc} \begin{array}{cc} -\omega_{a}^{2}\text{Re}\left[Z\left(\omega_{a}\right)\right] & -\omega_{a}\text{Im}\left[Z\left(\omega_{a}\right)\right]\\ -\omega_{a}^{2}\text{Im}\left[Z\left(\omega_{a}\right)\right] & \omega_{a}\text{Re}\left[Z\left(\omega_{a}\right)\right] \end{array} & \begin{array}{cc} \text{Re}\left[Z\left(\omega_{a}\right)\right] & -1\\ \text{Im}\left[Z\left(\omega_{a}\right)\right] & 0 \end{array}\\ \begin{array}{cc} -\omega_{b}^{2}\text{Re}\left[Z\left(\omega_{b}\right)\right] & -\omega_{b}\text{Im}\left[Z\left(\omega_{b}\right)\right]\\ -\omega_{b}^{2}\text{Im}\left[Z\left(\omega_{b}\right)\right] & \omega_{b}\text{Re}\left[Z\left(\omega_{b}\right)\right] \end{array} & \begin{array}{cc} \text{Re}\left[Z\left(\omega_{b}\right)\right] & -1\\ \text{Im}\left[Z\left(\omega_{b}\right)\right] & 0 \end{array} \end{array}\right)\left(\begin{array}{c} \begin{array}{c} m\\ b \end{array}\\ \begin{array}{c} k\\ F \end{array} \end{array}\right)=\left(\begin{array}{c} \begin{array}{c} 0\\ 0 \end{array}\\ \begin{array}{c} 0\\ 0 \end{array} \end{array}\right) \end{array} \end{array}$$ (4)

Given N=1, $\vec{p}\;$ comprises four unknown values: m,b,k, and F. We applied SVD to solve Eq. 4 for $\vec{p}$, with the scaled values and associated errors listed in Table 1. We propagated the errors in $\vec{p}\;$ by sampling from Gaussian mean distributions of the two phases, mean drive frequencies, and mean amplitudes to generate a set of Z. We then used SVD to solve for $\vec{p}$ corresponding to each Z, resulting in distributions of each output parameter (see the Supplementary Materials).

**Table 1.** Values of $\vec{p}$ from NetMAP and Unity LS for *N* = 1 cluster, scaled assuming *k* = 1 N/m.

Mechanical parameter	NetMAP	Unity LS
k(N/m)	1 (scaling factor)	1
$m\;(10^{-17}\;\mathrm{kg)}$	6.790±0.002	6.785
$b\;(10^{-11}\;\mathrm{kg/s)}$	2.388±0.643	2.283
$F\;(10^{-7}\;\mathrm{N)}$	8.713±0.286	8.107
$\mathrm{Amplitude}\;R^{2}$	0.98	0.97
$\mathrm{Phase}\;R^{2}$	0.97	0.99

To assess how accurately the resulting $\vec{p}\;$ characterized the single resonator, we compared the scaled values to expected values. The spring constant of suspended multilayer graphene (*30*) is ~1 to 5 N/m, so rescaling $\vec{p}\;$ by k=1 N/m provides order-of-magnitude estimates of all other parameters. We estimate the mass of the resonator using the area density of pristine graphene $\left(\rho =0.75\;\text{mg}/m^{2}\right)$ and an approximated area based on the suspended region highlighted in Fig. 2A, which gives a mass of $2.7\times 10^{-17}$ kg. While this estimate is lower than the value in Table 1, graphene contamination can increase the mass (*28*, *31*) by ~10×, placing the predicted m within the expected range. In addition, we estimated the damping of the resonator by fitting full amplitude spectra to a mass-normalized equation (see the Supplementary Materials). Using the fitted values, along with a value of k=1 N/m, we estimate the damping to be $b=1.92\times 10^{-11}$ kg/s, which agrees with the value in Table 1.

To evaluate the predictive power of the NetMAP results, we used $\vec{p}\;$ to calculate continuous analytical functions ∣Z(ω)∣ and ϕ(ω) and compare the results to the full experimental spectra. The $\vec{p}$-calculated predictions for ∣Z(ω)∣ and ϕ(ω) are shown as black dashed lines overlaid on the spectra (Fig. 2F) and the resulting $R^{2}$ (coefficient of determination) values are listed in Table 1. We emphasize again that the predicted ∣Z(ω)∣ and ϕ(ω) are uniquely determined. However, their calculation does not require the absolute values of $\vec{p}$; they can be obtained solely from the response vectors without the need for additional independent measurements. We see that, despite building the analytical spectra from just two data points, the predictions agree extremely well with the data, accounting for a minimum of 97% of variation in the spectral data. This highlights the remarkable efficiency of NetMAP, predicting a pair of nonlinear functions from just two data points and achieving excellent agreement with the full spectral response.

As additional validation, we compared NetMAP to nonlinear least-squares fitting with order-of-magnitude initial guesses (Unity LS). The fit parameter estimates obtained using Lmfit in Python are listed in Table 1; we have omitted the least-squares parameter error, but errors exceeded 3000% in some cases (see the Supplementary Materials). To compare the predicted $\vec{p}\;$ from each method, we use a two-tailed t test with the Unity LS parameters as reference values. We find that the values for damping b agree (p=0.87), and, while the mass m(p=0.004) and force F(p=0.03) do not agree, the mass from Unity LS still falls within the expected range and the force only differs by 7%. It is likely that including the Unity LS parameter variance would yield agreement under two-sample t tests. We also find that the $\vec{p}\;$ from Unity LS had similar predictive power when compared with the experimental spectra (see Table 1). We used NetMAP to characterize an additional N=1 cluster and attained similar results (see the Supplementary Materials). Our algebraic approach does not require a priori information and only uses two vector data points, as opposed to LS which uses thousands of spectral points and requires initial guesses, but we still observed agreement in the results of the two approaches for an N=1 resonator cluster. This demonstrates that NetMAP provides a highly efficient and practical alternative to traditional nonlinear least-squares regression, achieving comparable accuracy without requiring full spectra or the iterative guess-and-check process for initial parameter estimates. By extracting key system parameters with minimal input, NetMAP streamlines the characterization process, making it a powerful tool for studying nanomechanical resonators, even when isolated.

**Fig. 3.**
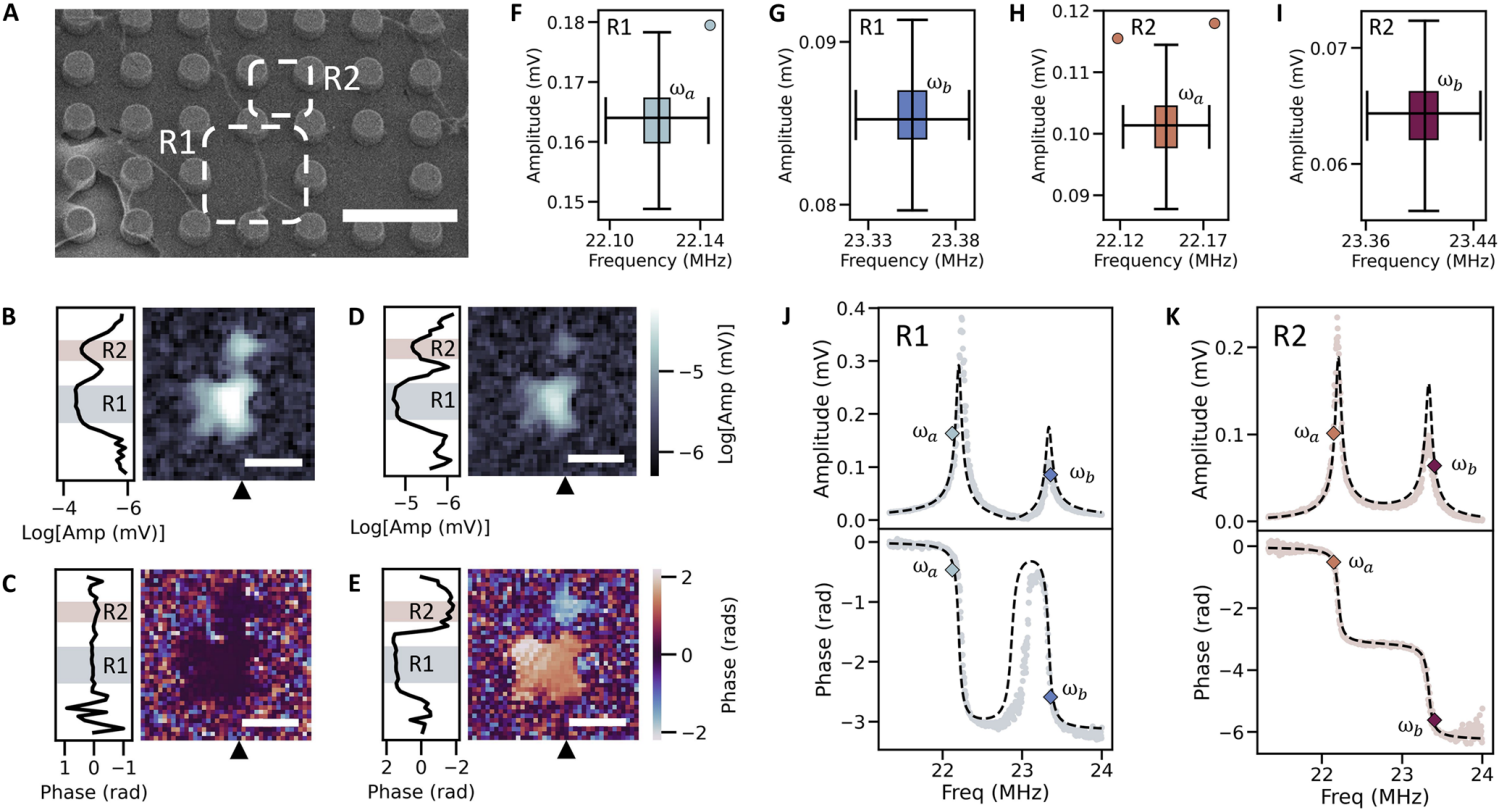
NetMAP analysis of N=2 cluster. (**A**) SEM image of driven resonator, R1, and neighboring coupled resonator, R2. Pillar radii are 0.75μm, and pillar pitch is 3μm. Scale bar, 6μm. (**B**) Amplitude (Amp) and (**C**) phase spatial maps taken at a drive frequency o$f\;f_{1}=21.51\;\text{MHz}$. Scale bars, 5μm. (**D**) Amplitude and (**E**) phase spatial maps taken at a drive frequency of $f_{2}=23.36\;\text{MHz}$. Scale bars, 5μm. Phase spatial maps (C) and (E) show uncorrected wrapped phase values. Black triangles on the bottom axes indicate the location of the vertical line scan on the left-side axis. 2D boxplots of PLL measurement distributions of frequency and amplitude for a phase lock of (**F**) $\phi_{1}\left(\omega_{a}\right)=-0.47\pm 0.06$ rad, (**G**) $\phi_{1}\left(\omega_{b}\right)=-2.59\pm 0.06$ rad, (**H**) $\;\phi_{2}\left(\omega_{a}\right)=-0.51\pm 0.2$ rad, and (**I**) $\phi_{2}\left(\omega_{b}\right)=-5.61\pm 0.2$. (**J**) Amplitude (top) and corrected phase (bottom) of R1. Diamond points in the amplitude plot correspond to PLL measurements of amplitude [$\bar{A_{1}}\left(\omega_{a}\right)=0.16368\pm 0.00005$ mV and $\bar{A_{1}}\left(\omega_{b}\right)=0.08560\pm 0.00003$ mV] and frequency (Freq) [$\bar{\Omega_{1}}\left(\omega_{a}\right)\;/\;2\pi =22.1208\pm 0.00008\;\text{MHz}$ and $\bar{\Omega_{1}}\left(\omega_{b}\right)\;/\;2\pi =23.3554\pm 0.0001\;\text{MHz}$]. Diamond points in the phase plot correspond to the phase lock values, $\phi_{1}\left(\omega_{a}\right)$ and $\phi_{1}\left(\omega_{b}\right)$, and the mean frequency values, $\bar{\Omega_{1}}\left(\omega_{a}\right)$ and $\bar{\Omega_{1}}\left(\omega_{b}\right)$. The black dotted lines represent $|Z_{1}\left(\omega \right)|$ and $\phi_{1}\left(\omega \right)$ generated from the normalized $\vec{p}$. (**K**) Amplitude (top) and corrected phase (bottom) of R2. Diamond points in the amplitude plot (top) correspond to PLL measurements of amplitude [${\bar{A}}_{2}\left(\omega_{a}\right)=0.10146\pm 0.00003$ mV and $\bar{A_{2}}\left(\omega_{b}\right)=0.06420\pm 0.00002$ mV] and frequency [$\bar{\Omega_{2}}\left(\omega_{a}\right)\;/\;2\pi =22.14721\pm 0.00007\;\text{MHz}$ and $\bar{\Omega_{2}}\left(\omega_{b}\right)\;/\;2\pi =23.4029\pm 0.0001\;\text{MHz}$]. Diamond points in the phase plot (bottom) correspond to the phase lock values, $\phi_{2}\left(\omega_{a}\right)$ and $\phi_{2}\left(\omega_{b}\right)$, and the mean frequency values, $\bar{\Omega_{2}}\left(\omega_{a}\right)\;/\;2\pi$ and $\bar{\Omega_{2}}\left(\omega_{b}\right)\;/\;2\pi$. The black dotted lines represent $|Z_{2}\left(\omega \right)|$ and $\phi_{2}\left(\omega \right)$ generated from the normalized $\vec{p}$.

We next increased the system degrees of freedom and tested NetMAP on a cluster size of N=2. The pair of coupled resonators are highlighted as R1 and R2 in the SEM in Fig. 3A, with SIM images showing the symmetric mode (Fig. 3, B and C), where R1 and R2 oscillate in phase, and the antisymmetric mode (Fig. 3, D and E), where R1 and R2 oscillate ~π radians out of phase (see the Supplementary Materials).

To measure $\omega_{a},\omega_{b},\vec{Z}\left(\omega_{a}\right)$, and $\vec{Z}\left(\omega_{b}\right)$ for the coupled pair, we chose a target value of $\omega_{a}$ below the symmetric mode peak and $\omega_{b}$ above the antisymmetric mode peak, shown for R1 in Fig. 3J (top) and for R2 in Fig. 3K (top). We used the corrected R1 phase spectra, Fig. 3J (bottom), to map the chosen $\omega_{a}$ and $\omega_{b}$ to phase values of $\phi_{1}\left(\omega_{a}\right)$ and $\phi_{1}\left(\omega_{b}\right)$. With the probe positioned over R1, we acquired PLL time-series measurements for the two phase values, shown as 2D boxplots in Fig. 3 (F and G). We repeated these PLL measurements for the second resonator by using the corrected R2 phase spectra, Fig. 3K (bottom), to map $\omega_{a}$ and $\omega_{b}$ to phase values of $\phi_{2}\left(\omega_{a}\right)$ and $\phi_{2}\left(\omega_{b}\right)$. We positioned the probe over R2, fixed the pump over R1, and acquired PLL time-series measurements for each phase, shown as 2D boxplots in Fig. 3 (H and I). We then used the mean values of amplitude, [$\bar{A_{1}}\left(\omega_{a}\right)$ and $\bar{A_{1}}\left(\omega_{b}\right)$], and frequency, [$\bar{\Omega_{1}}\left(\omega_{a}\right)$ and $\bar{\Omega_{1}}\left(\omega_{b}\right)$], to calculate $\omega_{a}=\left(\frac{1}{2}\right)\left[\bar{\Omega_{1}}\left(\omega_{a}\right)+\bar{\Omega_{2}}\left(\omega_{a}\right)\right]$ and $\;\vec{Z}\left(\omega_{a}\right)=\left\{\bar{A_{1}}\left(\omega_{a}\right)e^{i\phi_{1}\left(\omega_{a}\right)},\bar{A_{2}}\left(\omega_{a}\right)e^{i\phi_{2}\left(\omega_{a}\right)}\right\}$, and similarly to calculate $\omega_{b}$ and $\vec{Z}\left(\omega_{b}\right)$.

To solve for $\vec{p}\;$ of the N=2 cluster, we populated Z using the experimentally measured values of $\omega_{a}$, $\omega_{b}$, $\vec{Z}\left(\omega_{a}\right)$, and $\vec{Z}\left(\omega_{b}\right)$. For a cluster size N=2, $\vec{p}\;$ has eight unknown components, $\vec{p}=\left\{m_{1},m_{2},b_{1},b_{2},k_{1},k_{2},c_{1},F\right\}$, and Z is an 8 × 8 matrix. We applied SVD to solve Eq. 3 for $\vec{p}\;$ and list the scaled values in Table 2. Errors are calculated as described above for the N=1 cluster (see the Supplementary Materials). Scaling by $k_{1}=1$ N/m, we find that the intrinsic spring $k_{2}$ is also within the expected range. Assuming R1 and R2 have equal masses and intrinsic springs, we can estimate $c_{1}=0.05$ N/m (see the Supplementary Materials), which agrees with the value $c_{1}$ from Table 2. The damping constants $b_{1}$ and $b_{2}$ agree within error with each other and with the damping predicted above for the N=1 case, as expected because both clusters are on the same sample (*32*). In addition, based on the areas of R1 and R2, we predict the mass of each resonator to be $m_{1}=2.7\times 10^{-17}$ kg and $m_{2}=6.75\times 10^{-18}$ kg. Accounting for contamination, both $m_{1}$ and $m_{2}$ estimates in Table 2 are plausible. However, we note that NetMAP predicts $m_{1}<m_{2}$, whereas we expect $m_{1}>m_{2}$ because of the relative sizes of R1 and R2. We speculate that the mass discrepancy may be due to variations in local temperature from the optical probe position (*28*) and PLL-related error.

**Table 2.** Values of $\vec{p}$ from NetMAP and Unity LS for *N* = 2 cluster, scaled by *k*_1_= 1 N/m.

Mechanical parameter	NetMAP	Unity LS
$k_{1}\;\mathrm{(N/m)}$	1 (scaling factor)	1
$k_{2}\;\mathrm{(N/m)}$	1.784±0.044	1.655
$c_{1}\;\mathrm{(N/m)}$	0.069±0.002	0.057
$m_{1}(10^{-17}\;\mathrm{kg)}$	5.263±0.365	5.289
$m_{2}\;(10^{-17\;\mathrm{kg)}}$	8.975±0.219	8.165
$b_{1}\;(10^{-11}\;\mathrm{kg/s)}$	1.424±1.667	1.530
$b_{2}\;(10^{-11\;\mathrm{kg/s)}}$	5.405±4.625	4.846
$F\;(10^{-6\;\mathrm{N)}}$	1.501±0.651	1.358
$\mathrm{R1 amplitude}\;R^{2}$	0.73	0.89
$\mathrm{R1 phase}\;R^{2}$	0.83	0.99
$\mathrm{R2 amplitude}\;R^{2}$	0.85	0.85
$\mathrm{R2 phase}\;R^{2}$	0.996	0.996

To assess the predictive power of $\vec{p}\;$ for the coupled pair, we validated the analytical ${\vec{Z}}_{1}\left(\omega \right)$ and ${\vec{Z}}_{2}\left(\omega \right)$ against the full experimental spectra. The predicted analytical responses are shown as black dashed lines overlaid on the spectra in Fig. 3 (J and K) with the resulting $R^{2}$ values listed in Table 2. The NetMAP model predictions account for at least 73% of variability in the complete set of full experimental spectra ($R^{2}>0.73$ for all cases), with agreement as a high as 99.6% (e.g., R2 phase). We tested two additional pairs of coupled resonators (see the Supplementary Materials) and observed similar excellent agreement with the spectral response predicted by NetMAP. Although the predictions are strong, they may have been influenced by heating from the probe laser (*28*), which can locally modulate the elasticity of the resonators and introduce discrepancies in the data. We suspect that this heating effect contributes to the minor inconsistencies observed in Fig. 3J, particularly in the phase spectrum, where the predicted (dashed) curve does not fully capture the onset and width of the phase shift in the second peak. These discrepancies are likely due to the all-optical readout setup used in our experiment. They could be mitigated through heating calibration or by switching to an electronic readout. Despite the increased complexity of the hybridized modes compared to the single resonator case, NetMAP still produced remarkably accurate analytical predictions using only two spectral response vectors, a testament to its effectiveness.

To further evaluate our approach, we compare the NetMAP-predicted $\vec{p}\;$ with results from Unity LS (see Table 2). We find that the two approaches agree but only with precise order-unity a priori parameter estimates for LS. Using order-unity guesses informed by the NetMAP $\vec{p}\;$ (e.g., $m_{1}=m_{2}=10^{-17}\;$ kg) and omitting least-squares error (see the Supplementary Materials), the two approaches agree for $\;m_{1}\left(p=0.94\right)$, $b_{1}$ (p=0.95), $b_{2}$ (p=0.90), and F (p=0.83). Although the values disagree for $\;k_{2}\;\left(p=0.003\right)$, $c_{1}\;\left(p=0,t_{0}=7\right)$, and $\;m_{2}\;\left(p=0.0002\right)$, each value predicted by least squares was still within the expected range discussed above and only differed from the NetMAP $\vec{p}\;$ by at most 8 to 21%. We also found that least squares yielded higher $R^{2}$ values, but it used full spectra, whereas NetMAP was limited to only two spectral data points. In addition, the agreement between NetMAP and unity LS is extremely sensitive to the LS input guess solutions (see the Supplementary Materials) and increasing the number of variables increases the likelihood and size of error (*20*). Given that our approach is not sensitive to input guesses, NetMAP provides a robust means to obtain physically accurate network parameters without a priori knowledge of those parameters.

## DISCUSSION

The combination of SIM and algebraic parameter analysis in NetMAP provides a means to spatially map and quantify the mechanical elements of a resonator network and is, therefore, a powerful and vital tool for programming resonator networks for future applications.

The spatial information from SIM is crucial for NetMAP, as it provides the cluster size (N) and the lateral positions of each resonator, including the driven resonator, which are necessary for modeling the cluster. Spectroscopy alone lacks spatial resolution and cannot identify which specific resonators, among many, participate in the hybridized modes. Additionally, weak coupling is difficult or impossible to detect using the spectra alone (*23*). Although the resonator pairs analyzed in this study and in our previous work (*27*) were not strictly in the weak-coupling regime (*23*), we tested NetMAP on numerous weakly coupled pairs in a separate simulation-based study (*29*) and found excellent agreement, even in the extreme weak-coupling limit. It is likely that our pillar-based device architecture, where resonators are directly connected via graphene, inherently favors strong coupling due to graphene’s exceptionally high stiffness (*33*). SIM is valuable because it detects all resonators that oscillate resonantly within the cluster, regardless of coupling strength. Moreover, Z can be constructed directly from SIM amplitude and phase images, where $Z_{i}\left(\omega \right)$ is obtained from spatially averaging over a given resonator. This approach eliminates drift noise from PLL measurements but may reduce the SNR.

To program a cluster of nanomechanical resonators (see Fig. 1A), it is imperative to know the properties of each resonator node (i.e., $k_{n}$, $m_{n}$, $b_{n}$, and $c_{n}$) in its current state. For example, to configure a cluster into a phononic crystal (*10*, *34*), the node resonance frequencies ($\omega_{n}\equiv \sqrt{k_{n}/m_{n}\;}$) and coupling constants ($c_{n}$) must be precisely tuned. To tune $\omega_{n}$, it is essential to know the initial values of $k_{n}$ and $m_{n}$, particularly when the tuning method is irreversible, such as in the additive or subtractive tuning of resonator mass (*35*). Although $\omega_{n}$ can be determined through least-squares fitting, correlations in the model make it challenging to separate the individual values of $k_{n}$ and $m_{n}$. Without additional spatial information, the node properties become even harder to distinguish, thereby preventing tuning. Additionally, the coupling between resonators in the phononic crystal must be finely tuned. In simple N=2 systems, coupling strength is typically inferred from avoided-crossing signatures in gate-tuned spectrographs (*19*, *36*). However, this approach becomes impractical for larger systems (i.e., N≥3), as it cannot provide the spatial location of the spring analogs. In contrast, NetMAP directly quantifies normalized physical parameters of each node, including $k_{n}$, $m_{n}$, and $c_{n}$, and provides their spatial ordering. The normalized values of $\vec{p}\;$ can be scaled by measuring a single parameter, such as $k_{n}$ with atomic force microscopy (*30*). The absolute values of $\vec{p}\;$ are not required for programmability. Therefore, NetMAP enables the programming of our network platform for a wide range of applications, such on-demand tailored phononic crystals, defect engineering (*37*), and wave propagation control (*34*), among others.

While we have now applied NetMAP to 1D clusters with size N=1 and N=2, it can also be used to efficiently map larger, higher-dimensional clusters or even entire networks. In 1D, NetMAP requires a minimum of two response measurements (at $\omega_{a}$ and $\omega_{b}$) per resonator to assemble Z, which forms a 4N×4N matrix. The number of SVD-specific operations (*38*) needed to obtain $\vec{p}\;$ will scale efficiently with polynomial time, $O\left(N^{3}\right)$, based on the dimension of Z. This technique readily scales to networks in two and three dimensions and with more complex coupling beyond nearest-neighbor interactions. In such cases, depending on cluster geometry, more than two complex response vectors may need to be measured to solve for the unknown parameters. By using NetMAP to characterize each cluster, we can create a map of the entire network, consisting of neighboring clusters connected by coupling springs with $c_{n}=0$. With a complete view of the network, the edge $c_{n}$ values can be modified to form larger clusters or link clusters into a fully interconnected network.

Thus far, we have reported solutions to $Z\vec{p}=\vec{0}$ that belong to a 1D null space. However, the null space can be of higher dimension. When considering all potential singular values (λ≪1), the general solution would be a linear combination of the vectors corresponding to each λ, i.e., $\vec{p}=\sum \alpha_{i}{\vec{p}}_{i}$. The weights $\alpha_{i}$ can be determined by specifying additional constraints, such as estimating masses of each resonator. Alternatively, we could use least-squares to solve for $\alpha_{i}$ (see the Supplementary Materials). This approach would leverage the NetMAP results, avoiding the need to provide a priori information about the network parameters and offering an efficient way to account for higher-dimensional null spaces.

NetMAP’s algebraic characterization method can be applied to a variety of linear resonator networks, or any system that mathematically maps to such a network, as long as spatially resolved amplitude and phase responses for a cluster can be obtained, as we do here using SIM. These include alternative geometries of NEMS arrays (*12*, *39*), optical lattices (*3*), inductor-resistor-capacitor (LRC)-based electronic circuits, and biological networks. NetMAP could be extended to nonlinear networks by first using it to extract $\vec{p}$ under the assumption of linearity. The full spectra could then be fit with nonlinear regression, using $\vec{p}\;$ as initial guesses for the linear terms. This approach would isolate anharmonic contributions within a markedly reduced parameter space, with the linear elastic terms serving as upper bounds for the nonlinear terms. Moreover, a modified version of NetMAP will also be applicable to quantum analogs, such as chains of trapped ions (*1*) and 2D superconducting qubit arrays (*2*), and could be useful for programming the initial state for quantum computational tasks.

In conclusion, we demonstrate how NetMAP can spatially map and quantify all the physical parameters in local suspended graphene resonator clusters of sizes N=1 and N=2. By combining spatially resolved measurements of the resonator nodes and an algebraic solution of the network equations of motion, NetMAP offers a characterization method that addresses the limitations of current spectroscopic and regression-based techniques. This breakthrough offers a versatile modeling tool applicable across multiple disciplines and enables the development of programmable resonator networks. Such networks have the potential to drive applications like modeling natural systems, realizing mechanical computing schemes (*14*, *40*–*42*), interfacing with quantum information circuits (*43*), and exploring physics, including phononic metamaterials and exotic states (*12*, *15*, *34*).

## MATERIALS AND METHODS

### Fabricating pillar substrates

We created the suspended graphene resonator arrays by patterning SiO_2_/Si substrates using electron beam (e-beam) lithography followed by a dry reactive ion etch (CHF_4_), resulting in SiO_2_ pillars of ~600 nm in height. We began our fabrication process with a doped Si wafer with 1 μm of commercially grown oxide. To prepare the sample for lithography, we sonicated the diced wafer in acetone and then IPA, followed by a deionized water (DI) rinse and N_2_ dry. The sample was then dehydrated on a hotplate at $400^{\circ }$C for 30 min before spinning on poly(methyl methacrylate) (PMMA) A4 to improve the adhesion of the resist. We used PMMA A4 resist with a thickness of 200 nm that was soft baked at $180^{\circ }$C for 1 min to evaporate the PMMA solvent. We patterned dot arrays, 0.25 to 0.75 μm in radius and 1 to 4.5 μm in pitch, into the resist using e-beam lithography with nanometer pattern generation system (NPGS) on a Zeiss scanning electron microscope. In these arrays, we varied the size of the resonators by omitting specific dots and varied the potential coupling strength with one or two rows of pillars separating the designated resonators. To remove the resist from the exposed dot regions, we developed the sample with MIBK 3:1 for 1 min. We then deposited a 20- to 30-nm Cr mask with thermal evaporation (Angstrom Engineering Amod) followed by a PMMA liftoff in Remover PG on a hotplate at $50^{\circ }$C for 1 hour. The remaining Cr now covered only the dot regions that were previously exposed with e-beam lithography. To form the pillars, we used CHF_4_ in an inductively coupled plasma instrument to etch the exposed regions of SiO_2_ that were not masked with Cr, resulting in pillars that were 600±50 nm. To prevent electrical shorting, we left ~400 nm of oxide as an insulating layer, such that any collapsed graphene would not contact the conducting Si directly. Last, we etched the remaining Cr to leave only the SiO_2_ pillars on silicon. Additionally, we omit pillars throughout the array to create larger-size resonators and resonator pairs (Fig. 1, C and E). The lateral size of the membrane resonators varies from 3 to 6 μm. The resonators are directly connected by suspended graphene, which provides a mechanism for elastic coupling represented by the coupling spring constants, $c_{n}$, in Fig. 1A.

### Graphene transfer method

We then suspended commercially grown CVD graphene over the pillar arrays using a wet transfer method (*44*). To protect the graphene during the transfer, we spun 200 nm of PMMA A4 onto the graphene, resulting in a Cu/Graphene/PMMA sheet. We then used ammonium persulfate (40 mg/ml) to etch the copper, leaving only the graphene/PMMA film. We rinsed the graphene/PMMA film in multiple water baths to remove any ammonium persulfate and then used the pillared sample to scoop the film from below, such that the graphene was in direct contact with the sample and the PMMA on top of the graphene. We let the sample dry overnight and then baked the sample at $120^{\circ }$C for 5 min to aid the adhesion between the graphene and the substrate. To remove the PMMA, we let the sample soak in acetone for 4 to 6 hours. To avoid increased tension in the membrane that could cause the membrane to tear, we removed the sample from solution using a critical point dry. The result was a continuous graphene film suspended across pillar arrays to create resonators that could be coupled directly through strain.

### Spectra measurement procedure

The first step needed to obtain the measurements needed to construct Z is to determine which drive frequencies (ω) provide a large response signal $|Z_{n}\left(\omega \right)|$, which we achieve by measuring amplitude and phase spectra of the *n*th resonator and searching for resonance peaks (Fig. 1D, step 1). To measure the spectra, we position a focused optical pump (445 nm) and a probe (633 nm) laser on the *n*th resonator. While modulating the pump with an acousto-optic modulator (AOM), we measure the corresponding amplitude and phase via interferometry and lock-in amplification (see Fig. 1, C, E, and F). All measurements are taken in vacuum ($∼\;10^{-7}\;\text{torr}$) at room temperature. From the resulting spectra, we locate resonance peaks to determine a range of frequencies that provide a large $|Z_{n}\left(\omega \right)|$. To further increase the signal, we increase the amplitude of the pump laser until $|Z_{n}\left(\omega \right)|$ is just below the limit of the linear regime. For all further measurements and steps, we fix the coordinates of the pump at this initial position.

### SIM measurement procedure

Second, we quantify the size N and spatial configuration of the cluster by obtaining images of the mechanical motion of the area surrounding the driven resonator SIM (*24*). For SIM, we modulate the fixed pump at a value of ω, chosen to correspond to a large signal in the measured amplitude spectrum. We then raster the probe across the sample while recording both amplitude and phase, resulting in 2D spatial images ∣Z(ω,x,y)∣ and ϕ(ω,x,y) (Fig. 1D, step 2). SIM images are taken over a large enough area to characterize the local vicinity of the driven resonator, typically about 20μmby20μm in size. To quantify N, we use ∣Z(ω,x,y)∣ and ϕ(ω,x,y) to count the number of resonators in the cluster, corresponding to the number of regions that have local amplitude maxima and constant phase. To confirm N and map the spatial configuration of the cluster, we cross-correlate ∣Z(ω,x,y)∣ with an optical bright-field (Fig. 1F) and SEM (Fig. 1B) image to match peak amplitudes with specific areas of the suspended membrane. Our homebuilt SIM software allows for easy point-and-click positioning of the probe, which we use to collect spectra (step 1) for each resonator in the cluster. With a full set of spectra, we determine which frequencies ω result in a largest overall response signal. Moreover, we use the point-and-click positioning feature to finely tune the x-y probe coordinates over each resonator to further maximize the signal.

### PLL measurement procedure

The third and final step is to measure the components of Z, constructed with $\;\omega_{a},\omega_{b},$ $\vec{Z}\left(\omega_{a}\right),\text{and}\;\vec{Z}\left(\omega_{b}\right)$. To obtain the first set $\left[\omega_{a},\vec{Z}\left(\omega_{a}\right)\right]$, we position the probe on the *n*th resonator, but rather than fixing $\omega_{a}$ and measuring $Z_{n}\left(\omega_{a}\right)$, we use a PLL to monitor the time series of frequency and amplitude, $\Omega_{n}\left(\omega_{a}\right)$ and $A_{n}\left(\omega_{a}\right)$, for a fixed phase input of $\phi_{n}\left(\omega_{a}\right)$. The phase input is determined by the value of the phase spectrum at the target $\omega_{a}$. We record each time series until the amplitude SNR reaches a predetermined value of $\sim 10^{3}$, typically resulting in $\sim \;10^{4}$ discrete measurements. We define the SNR as the ratio of the mean of the series to the SE ($|\bar{A}|\;/\;\sigma_{\bar{A}}$). Although the best choice of a target $\omega_{a}$ for high SNR would be at the peak resonance, we chose frequencies slightly off resonance to reduce error from frequency-dependent linear phase lags intrinsic to the optical set up (see the Supplementary Materials for details). Additionally, the phase must be corrected for this lag before calculating $Z_{n}\left(\omega_{a}\right)$. We correct the phase by linearly fitting a region of the phase spectrum far off resonance to determine an intercept, $\phi_{0}$, and the time delay slope, τ. We then subtract the quantity $\;\phi_{0}-\omega_{i}\tau$ from the PLL input phase (see the Supplementary Materials for details). After repeating this PLL measurement for all N resonators in the cluster, we calculate $\left[\omega_{a},\vec{Z}\left(\omega_{a}\right)\right]$ as $\omega_{a}=\sum_{n=1}^{N}\frac{{\bar{\Omega }}_{n}\left(\omega_{a}\right)}{N}$ and $\vec{Z}\left(\omega_{a}\right)=\left\{\bar{A_{1}}e^{i\phi_{1}\left(\omega_{a}\right)},\;\ldots ,\bar{A_{N}}e^{i\phi_{N}\left(\omega_{a}\right)}\right\}$, in which we use the corrected phase values, $\phi_{n}\left(\omega_{a}\right),$ and the time series averages, ${\bar{\Omega }}_{n}\left(\omega_{a}\right)$ and $\bar{A_{n}}\left(\omega_{a}\right)$. We repeat this procedure to calculate $\left[\omega_{b},\vec{Z}\left(\omega_{b}\right)\right]$, completing all the measurements needed to calculate the components of Z.

## Supplementary Materials

### This PDF file includes:

Supplementary Text Figs. S1 to S6 Tables S1 to S3 References
