# Supplementary material for: A minimal-data approach for spatially resolved parameter analysis of coupled graphene nanomechanical resonators
Source: Sci Adv. 2025 Nov 14;11(46):eadq0621. doi: 10.1126/sciadv.adq0621 (PMC13141894; doi:10.1126/sciadv.adq0621)
Supplement: Supplementary file 1 — Supplementary Text Figs. S1 to S6 Tables S1 to S3 References [file sciadv.adq0621_sm.pdf]

Science Advances
AAAS

Supplementary Materials for

A minimal-data approach for spatially resolved parameter analysis of coupled graphene nanomechanical resonators

Brittany Carter *et al.*

Corresponding author: Benjamín J. Alemán, baleman@uoregon.edu

Sci. Adv. **11**, eadq0621 (2025)
DOI: 10.1126/sciadv.adq0621

This PDF file includes:

Supplementary Text
Figs. S1 to S6
Tables S1 to S3
References

Supplementary Text

Section S1: Algebraic approach

The linear mass and spring model is described by an infinite set of coupled differential equations.

$$
\begin{gathered}
\vdots \\
m_{i-1}\ddot{x}_{i-1} + b_{i-1}\dot{x}_{i-1} + k_{i-1}x_{i-1} + c_{i-1}(x_{i-1} - x_i) + c_{i-2}(x_{i-1} - x_{i-2}) = F_{i-1}\mathrm{e}^{\mathrm{i}\omega\mathrm{t}} \\
m_i\ddot{x}_i + b_i\dot{x}_i + k_ix_i + c_{i-1}(x_i - x_{i-1}) + c_i(x_i - x_{i+1}) = F_i\mathrm{e}^{\mathrm{i}\omega\mathrm{t}} \\
m_{i+1}\ddot{x}_{i+1} + b_{i+1}\dot{x}_{i+1} + k_{i+1}x_{i+1} + c_i(x_{i+1} - x_i) + c_{i+1}(x_{i+1} - x_{i+2}) = F_{i+1}\mathrm{e}^{\mathrm{i}\omega\mathrm{t}} \\
\vdots
\end{gathered}
$$

This system of equations can be organized into the matrix form

$$\mathbf{M}\ddot{\vec{\mathrm{x}}} + \mathbf{B}\dot{\vec{\mathrm{x}}} + \mathbf{K}\vec{\mathrm{x}} = \vec{\mathrm{F}}\,\mathrm{e}^{\mathrm{i}\omega\mathrm{t}} \tag{S1}$$

where the three matrices are

$$
\boldsymbol{M} = \begin{pmatrix} \ddots & 0 & 0 & 0 & \iddots \\ 0 & m_{i-1} & 0 & 0 & 0 \\ 0 & 0 & m_i & 0 & 0 \\ 0 & 0 & 0 & m_{i+1} & 0 \\ \iddots & 0 & 0 & 0 & \ddots \end{pmatrix}, \quad
\mathbf{B} = \begin{pmatrix} \ddots & 0 & 0 & 0 & \iddots \\ 0 & b_{i-1} & 0 & 0 & 0 \\ 0 & 0 & b_i & 0 & 0 \\ 0 & 0 & 0 & b_{i+1} & 0 \\ \iddots & 0 & 0 & 0 & \ddots \end{pmatrix},
$$

$$
\mathbf{K} = \begin{pmatrix} \ddots & -c_{i-2} & 0 & 0 & \iddots \\ -c_{i-2} & k_{i-1} + c_{i-2} + c_{i-1} & -c_{i-1} & 0 & 0 \\ 0 & -c_{i-1} & k_i + c_{i-1} + c_i & -c_i & 0 \\ 0 & 0 & -c_i & k_{i+1} + c_i + c_{i+1} & -c_{i+1} \\ \iddots & 0 & 0 & -c_{i+1} & \ddots \end{pmatrix}.
$$

To solve Eq (*S1*), we use a steady-state trial solution of $x_i(t) = Z_i(\omega)e^{i\omega t}$, where $Z_i(\omega) = A_i(\omega)e^{i\phi_i(\omega)}$ is the complex amplitude of the ith resonator. The phase $\phi_i(\omega)$ corresponds to the purely mechanical steady-state phase difference between the driving force and the response $A_i(\omega)$. We then insert this solution into Eq (*S1*) and obtain

$$-\omega^2\mathbf{M}\vec{\mathrm{Z}} + \mathrm{i}\omega\mathbf{B}\vec{\mathrm{Z}} + \mathbf{K}\vec{\mathrm{Z}} = \vec{\mathrm{F}} \tag{S2}$$

where the complex amplitude vector and force amplitude vector are defined as

$$
\vec{Z}(\omega) = \begin{pmatrix} \vdots \\ Z_{i-1}(\omega) \\ Z_i(\omega) \\ Z_{i+1}(\omega) \\ \vdots \end{pmatrix}, \quad \vec{F} = \begin{pmatrix} \vdots \\ F_{i-1} \\ F_i \\ F_{i+1} \\ \vdots \end{pmatrix}
$$

More compactly, the equation of motion can be written

$$\boldsymbol{\mathcal{M}}(\omega)\vec{Z}(\omega) = \vec{F} \quad \textbf{(S3)}$$

The symmetric, tridiagonal matrix $\boldsymbol{\mathcal{M}}(\omega) = -\omega^2 \boldsymbol{M} + i\omega\boldsymbol{\Gamma} + \boldsymbol{K}$ contains complete information about the inertia, elasticity, and damping of the network. Because the goal of our technique is to calculate the variables in $\boldsymbol{\mathcal{M}}(\omega)$ by measuring $\vec{Z}(\omega)$, we reorganize a finite version of Eq (*S3*) as a homogenous linear equation

$$\boldsymbol{\mathcal{Z}}\vec{p} = \vec{0} \quad \textbf{(S4)}$$

In Eq (S4), $\vec{p}$ consists of all the unknown mechanical parameters and $\boldsymbol{\mathcal{Z}}$ is a real valued matrix with components calculated from the set drive frequency ω and the real and imaginary components of $\vec{Z}(\omega)$.

For a cluster of size N, the number of unknown parameters in $\vec{p}$ is $4N$; with N masses, N damping constants, N intrinsic springs, $N-1$ coupling springs, and 1 force (assuming one driven resonator). Each measurement of $\vec{Z}(\omega)$ accounts for $2N$ rows in $\boldsymbol{\mathcal{Z}}$, due to the real and imaginary part of each resonator's equation of motion. Therefore, to solve for all the parameters in $\vec{p}$, we will need to take measurements of $\vec{Z}(\omega)$ for a minimum two drive frequencies, such that we have solve $4N$ equations to solve for $4N$ unknowns. We can incorporate measurements taken at additional drive frequencies by using the same SVD package to solve the overdetermined system of equations. In certain cases, the amplitude of some resonators at a given ω will be "zero", or undetectable by the apparatus. When a resonator's motion is undetectable, the number of linear equations is reduced; if the undetectable resonator is in the interior of the cluster, six equations are lost, while if at the edge of the cluster four equations are lost. Therefore, it is important to ensure that all resonators have a measurable amplitude for two or more driving frequencies.

Section S2: Experimental Phase lag

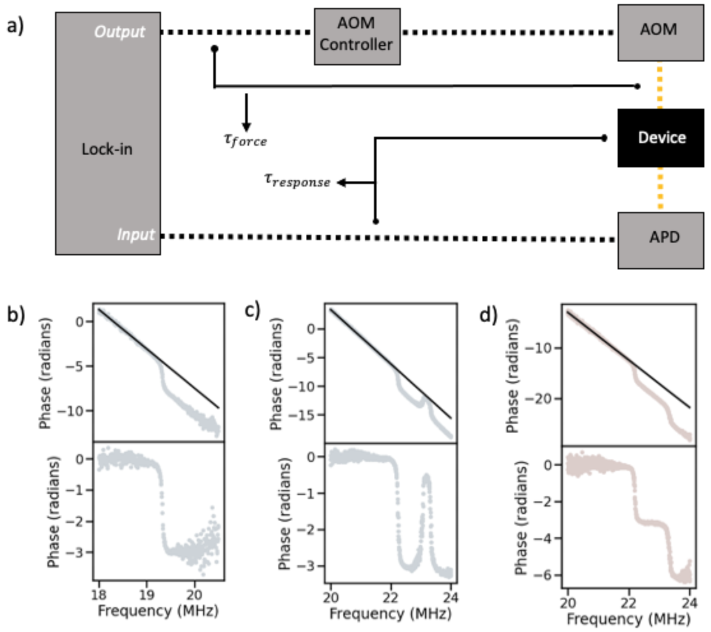

Figure S1: Experimental phase lag.
a) Diagram of the experimental time delay phase shift. Time delay, τ_{force}, includes delays due to transmission cables, shown as black dashed lines, the AOM controller, the AOM, and the free-space optical path, shown as yellow dashed lines. The phase shift on the response, τ_{response}, includes delays due to the free-space optical path, APD, and transmission cables. The total time delay is the sum $\tau = \tau_{\text{force}} + \tau_{\text{response}}$. **b)** Phase lag correction for $N = 1$ resonator. Upper plot shows uncorrected phase as gray data points and linear fit as black line, with $\tau = 700 \pm 7$ ns and $\phi_0 = 80.5 \pm 0.8$ rad. Lower plot shows corrected phase as gray data points. Phase lag corrections for $N = 2$ for **c)** R1 with upper plot showing uncorrected phase values as gray data points and linear fit as black line, with $\tau = 752 \pm 1$ ns and $\phi_0 = 97.8 \pm 0.1$ rad. Lower plot shows corrected phase values as gray data points and **d)** R2 with upper plot showing uncorrected phase values as light orange data points and linear fit as black line, with $\tau = 747 \pm 4$ ns and $\phi_0 = 90.9 \pm 0.5$ rad. Lower plot shows corrected phase values as light orange data points.

Prior to using the experimental phases to populate $\mathcal{Z}$, we first accounted for frequency-dependent phase lags caused by time delays of the optical and electronic signal transmission/transduction from the lock-in reference output to the input **(Figure S1a)**. The time-delay phase shift on the pump (i.e the force), τ_{force}, is the sum of delays due to the AOM controller, the AOM, and the free-space optical path and transmission cables up to the sample, shown in **Figure S1a**. The total time delay at the lock-in input includes τ_{force} and τ_{response}, which includes delays due to the photodetector, an "on-chip" thermal lag(*24*), and the free-space optical path and transmission cables from the device to the lock-in, see **Figure S1a**. There is also a constant phase lag due to the phase offset of the lock-in and a π phase lag that may arise depending on whether the membrane moves away from or toward the focusing objective(*24*). These time delays and offsets will result in a frequency-dependent phase shift according to:

$$\Delta(\omega) = \phi_0 - \omega\tau \tag{S5}$$

Where τ is the total frequency dependent time delay and ϕ_0 is the total constant phase offset. The result of this phase lag is evident in the phase data as a linear offset, $\phi_{\text{meas}}(\omega)$, shown as light gray or light orange data points in **Figure S1 b-d upper.** We obtain τ and ϕ_0 by fitting to a linear section of the raw phase spectra, shown as black solid lines in **Figure S1b-d upper.** We then correct for the phase lag by subtracting $\Delta(\omega)$ from the measured phase $\phi_{\text{meas}}(\omega)$

$$\phi(\omega) = \phi_{\text{meas}}(\omega) - \Delta(\omega) \tag{S6}$$

The corrected phase spectra, $\phi(\omega)$, are shown as light gray or light orange data points in **Figure S1b-d lower.** To account for spatial variations, we perform this calculation for each individual resonator in the cluster. For each measured resonator, we calculated t_{delay} to be $\sim 700 - 750$ ns, which is consisted with our estimate based on the optical components, lengths of free space optics, and lengths of electrical wiring in our set up. We calculate the propagated error in $\phi(\omega)$ by estimating the uncertainty(*45*) in the fitted linear section of $\phi_{\text{meas}}(\omega)$ using

$$\sigma_\phi = \sqrt{\frac{1}{N-2}\sum_{i=1}^{N}(\phi_i - \phi_0 - \tau\omega_i)^2} \tag{S7}$$

In addition to correcting the phase prior to populating $\boldsymbol{\mathcal{Z}}$, we also considered the experimental lags when choosing target values of ω_a and ω_b for PLL. The best choices of ω_a and ω_b for high SNR would be at the highest amplitude signals, which occur at peak resonance. However, the peak amplitude at resonance will also correspond to the region of phase with the largest slope. Because we lock into uncorrected phase values, the PLL data will be most affected by the phase lag for values near the steepest slope. We therefore choose target values of ω_a and ω_b to be slightly off resonance for the PLL measurements. Future experiments could achieve reduced error with a PLL that accounts for frequency dependent phase lags by locking onto a maximum slope or onto corrected phase values.

Section S3: Errors in parameter vector values

To assess the precision of NetMAP, we solve for $\vec{p}$ with a distribution of $\boldsymbol{\mathcal{Z}}$ matrices and calculate the standard deviation of each output parameter. For the single resonator cluster, we randomly sampled ω_i and $Z_1(\omega_i)$, for $(i = a, b)$, from normal distributions with a standard deviation equal to the standard error of the PLL time series $\Omega_1(\omega_i, t)$ and $|Z_1(\omega_i, t)|$. We randomly sampled $\phi_1(\omega_i)$ from a normal distribution with the standard deviation equal to the uncertainty calculated in equation Eq (*S7*)**.** We then used these randomly sampled values to populate $\boldsymbol{\mathcal{Z}}$ and applied SVD to solve for $\vec{p}$. We repeated this procedure $n = 1000$ times to obtain distributions of each output parameter (m, b, F). For the $N = 1$ cluster, we always scaled the parameters to the spring constant k, so this method did not provide an error for the spring constant. We then removed all solutions that were unphysical or deficient, i.e solutions in which the output parameters did not have a homogenous sign or the R^2 values were negative, and were left with distributions of $n = 859$ for each output parameters, with the results shown in **Figure S2a-c**. We quote the standard deviation of each distribution as the error on the parameter value in **Table 1** of the main text. To assess the predictive power of each output parameter set, we used each output $\vec{p}$ to calculate $\overrightarrow{Z_n}(\omega)$

and compared the result to the experimental spectra of amplitude and phase to calculate distributions of the R^2 values, shown in **Figure S2d**.

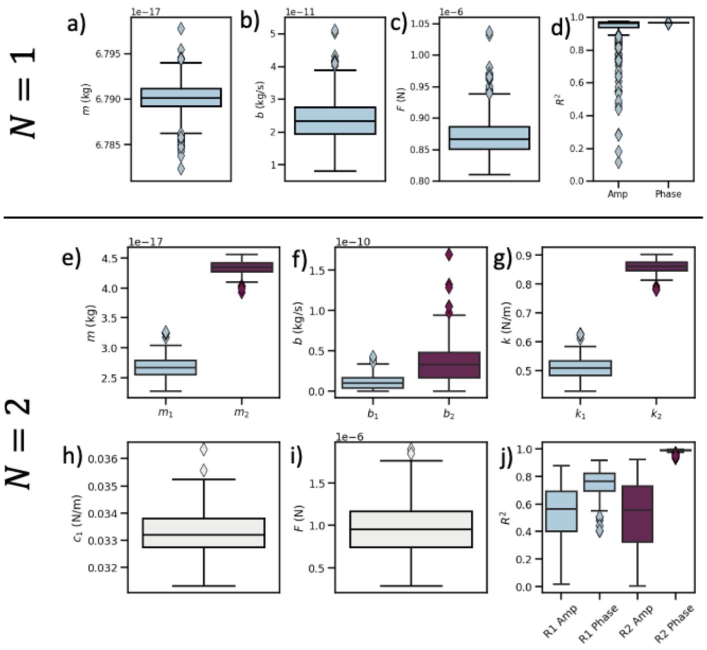

Figure S2: Parameter error distributions.
Error distributions as box and whisker plots showing median, upper, and lower quartile ranges with whiskers that extend to include 1.5 IQR. Quoted errors are standard deviations of each distribution. Distributions for $N = 1$ cluster parameters shown as light blue boxes with **a)** $m = (6.790 \pm 0.001) \times 10^{-17}$ kg , **b)** $b = (2.4 \pm 0.6) \times 10^{-11}$ kg/s, **c)** $F = (8.7 \pm 0.3) \times 10^{-6}$ N. **d)** $N = 1$ cluster distributions of R^2 values for amplitude ($R^2 = 0.94 \pm 0.08$) and phase ($R^2 = 0.968 \pm 0.001$). Distributions for $N = 2$ cluster with R1 distributions shown as light blue boxes and R2 distributions shown as maroon boxes for **e)** $m_1 = (5.2 \pm 0.4) \times 10^{-17}$ kg and $m_2 = (8.5 \pm 0.2) \times 10^{-17}$ kg, **f)** $b_1 = (2.4 \pm 1.9) \times 10^{-11}$ kg/s and $b_2 = (6.7 \pm 4.5) \times 10^{-11}$ kg/s, **g)** $k_1 = 1.00 \pm 0.08$ N/m and $k_2 = 1.69 \pm 0.05$ N/m. Error distributions for $N = 2$ cluster of **h)** $c_1 = 0.065 \pm 0.002$ N/m and **i)** $F = (1.9 \pm 0.7) \times 10^{-6}$ N. **j)** $N = 2$ cluster distributions of R^2 values with R1 distributions shown as light blue boxes for amplitude ($R^2 = 0.51 \pm 0.20$) and phase ($R^2 = 0.75 \pm 0.08$). R2 distributions are shown as maroon boxes for amplitude ($R^2 = 0.51 \pm 0.25$) and phase ($R^2 = 0.988 \pm 0.008$).

We repeated this procedure to calculate the errors for the output parameters of the $N = 2$ cluster. We produced a normal distribution to randomly sample ω_i with a standard deviation equal to standard errors of the R1 and R2 frequency time series, $\Omega_1(\omega_i, t)$ and $\Omega_2(\omega_i, t)$, added in quadrature. We randomly sampled the amplitudes and phases for each resonator, $Z_1(\omega_i)$ and $Z_2(\omega_i)$, using the same procedure described for the $N = 1$ cluster above. We then use these randomly sampled values to populate $\boldsymbol{\mathcal{Z}}$, used SVD to solve for $\vec{p}$, and repeated the procedure $n = 5000$ times. We then removed all non-physical solutions, resulting in distributions of $n = 253$ shown for each parameter $(m_1, m_2, b_1, b_2, k_1, k_2, k_{12}, F)$ in **Figure S2e-i**. We again assessed the predictive power of each output $\vec{p}$ by comparing calculated $\overrightarrow{Z_n}(\omega)$ and to the experimental spectra of the R1 and R2 amplitude and phase to obtain distributions of R^2 values, shown in **Figure S2j**.

Section S4: Further details for N=1 cluster in main text

To measure and confirm the size of the $N = 1$ cluster, we first positioned the pump and probe over the resonator region highlighted in **Figure 2a,** and acquire amplitude and phase spectra. The resulting spectra revealed a single peak in the amplitude, shown as grey data points in **Figure 2f (upper)**, that corresponded to a corrected phase of $\pi/2$ (**Figure 2f (lower)**, grey)**,** consistent with a single, uncoupled resonator. To confirm the size of this cluster was $N = 1$, we took a SIM scan at 18.81 MHz, resulting in the amplitude and phase spatial maps shown in **Figure 2b.** In the amplitude map, we observed an amplitude maximum ($\sim 10^{-4}$ mV) within one localized $\sim 6 \times 6\ \mu m^2$ region of the suspended graphene, which matched the size and location of the region highlighted in **Figure 2a**. The spatial undulations in the amplitude and phase near the edge of the resonator region are likely due to interactions with the pillars. Outside of the resonator region, the amplitude decreases by more than two orders of magnitude ($\sim 10^{-6}$ mV). Moreover, the resonator region has nearly constant phase ($STD = 0.07$ rad), implying it moves in unison, as expected for the fundamental mode of a single resonator. Away from the resonator, the phase is noisier (test statistic $F_0 \sim 74,\ p \sim 10^{-11}$), with an increase in the standard deviation by almost an order of magnitude ($STD = 0.6$ rad). Lastly, as seen in the line scans (**Figure 2b,c**), the amplitude has one solitary lobe with constant phase. Altogether, we conclude that this local cluster consists of a single, uncoupled resonator.

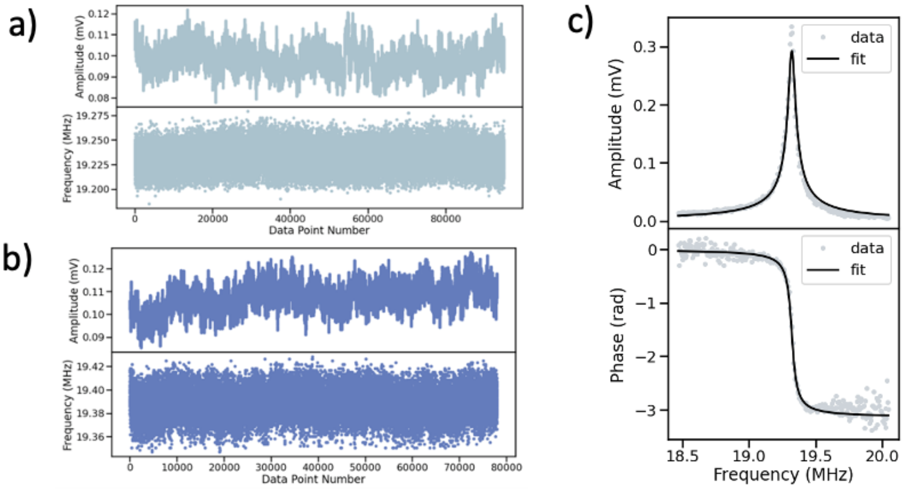

Figure S3: Additional figures for $N = 1$ cluster.
PLL measurements of amplitude and frequency plotted by data point number for corrected phase lock values of **a)** $\phi_1(\omega_a) = -0.33$ rad shown in light blue and **b)** $\phi_1(\omega_b) = -2.78$ rad shown in dark blue. **c)** least square fit result shown as solid black line over spectra data of amplitude (upper) and phase (lower) shown as gray data points.

To evaluate the stability of the PLL measurements over time, we replot the data that is displayed as 2D boxplots in **Figure 2d,e**, as a function of data point number. The PLL measurements of amplitude (upper) and frequency (lower) are plotted for phase lock values of $\phi_1(\omega_a)$, **Figure S3a**, and $\phi_1(\omega_b)$, **Figure S3b.** We observe that the frequency does not drift significantly in either PLL measurement. We do observe a slight increase in amplitude in the $\phi_1(\omega_b)$ PLL measurement, **Figure S3b upper**, which could be due to a shift up in the resonance frequency due to heating.

In the main text, we evaluate the accuracy of the NetMAP calculated $\vec{p}$ by comparing each calculated value to expected values. To estimate the expected value of the damping, b, we fit the amplitude spectra to a built-in Lmfit model, DampedOscillatorModel, which has three fit parameters: A, ω_0, and σ. Because there are low correlations between each parameter, the resulting values have low error. From the model equation and fit parameters σ and ω_0, we estimate the damping based on

$$2\sigma\omega_0 = \frac{b}{m}$$

We divide both sides by $\omega_0^2 = \frac{k}{m}$, where ω_0 is the fitted center frequency to obtain

$$b = \frac{2k\sigma}{\omega_0}$$

With fitted values of $\omega_0 = 2\pi \times (19.32 \text{ MHz})$ and $\sigma = 1.16 \times 10^{-3}$, along with the approximated value of $k = 1$ N/m, we estimate the damping to be $b = 1.92 \times 10^{-11}$ kg/s.

To further validate NetMAP, we compare the NetMAP output parameters to those from least-squares fitting with unity order of magnitude guesses, Unity LS. The fit result of Unity LS, listed in **Table 1** in the main text, is shown in **Figure S3c** as a solid black line overlayed on the experimental spectra. To compare each output parameter from NetMAP and LS, we perform a two tailed t-test, using the LS values as reference because the LS errors for each parameter value were large (up to 3000%). We suspect these large errors are due to the high correlations of multiple parameter pairs in our model. We also compared values by using a percent difference, which we calculate by taking the absolute value of the difference between the two values to be compared and dividing by the smaller of the two values for an upper bound estimate.

Section S5: Further details for $N = 2$ cluster in main text

To measure and confirm the size of the $N = 2$ resonator described in the main text, we positioned the pump and probe over the resonator region highlighted as R1 in **Figure 3a** and acquire amplitude and phase spectra. The resulting amplitude spectrum (**Figure 3f (upper), grey data points)** revealed two closely spaced peaks, each corresponding to a corrected phase of $\sim\pi/2$ (**Figure 3f (lower)**, grey), indicative of the two hybridized modes of a pair of coupled resonators(*23*). To test if the spectral features correspond to a resonator pair, we took a SIM scan across the region at a frequency below the first peak ($f_1 = 21.51\ MHz$). The resulting spatial maps (**Figure 3b,c**) show two distinct high-intensity amplitude regions with nearly constant phase, as highlighted in the line profiles (**Figure 3f)**. The first high-amplitude region was centered in a $6 \times 6\ \mu m^2$ region, which matched the size and location of the driven resonator R1 shown in **Figure 3a**. The second region matched the size and location of a $3 \times 3\ \mu m^2$ membrane highlighted as R2 in **Figure 3a**. The mean phases of R1 and R2 differed by 0.12 rad, or ~6.9°, (p~0.001), indicating they move in near unison, in accord with expectations for the symmetric mode of a coupled pair(*24*). We search for the asymmetric mode with an additional SIM scan at a frequency above the second spectral peak ($f_2 = 22.55\ MHz$). In the resulting amplitude map and cross-section (**Figure 3d**), we see two regions with high amplitude situated at the same locations as R1 and R2 in **Figure 3a,b**. The phase map and profile (**Figure 3e**) reveal the phase in each region is relatively

uniform, but the regions differ from each other by $\sim\pi$ rad, as expected for the asymmetric mode. From the spectra and SIM data, we conclude that the R1 and R2 resonators form a coupled pair.

To further support our conclusion that the spatial maps of amplitude and phase in **Figure 3b-e**, depict a $N = 2$ cluster, we compared the R1 and R2 regions, highlighted in **Figure 3a**, to surrounding areas of low amplitude and noisy phase. In the first SIM scan, **Figure 3b,c**, we observe a decrease in amplitude by more than an order of magnitude outside of the R1 and R2 resonator regions. Moreover, the R1 and R2 regions has nearly constant ($STD = 0.03$ rads, $STD = 0.05$ rads, respectively), whereas the phase outside the resonator regions is noisier ($STD = 0.57$), implying that the R1 and R2 regions each move with constant phase and notable amplitude. For the second SIM scan, **Figure 3d,e**, the amplitude decreased by more than an order of magnitude for R1 and by more than 70% for R2 outside of the two resonator regions. Outside of the resonator regions, the phase was also noisier with about a 70% increase in the standard deviation ($STD = 0.42$).

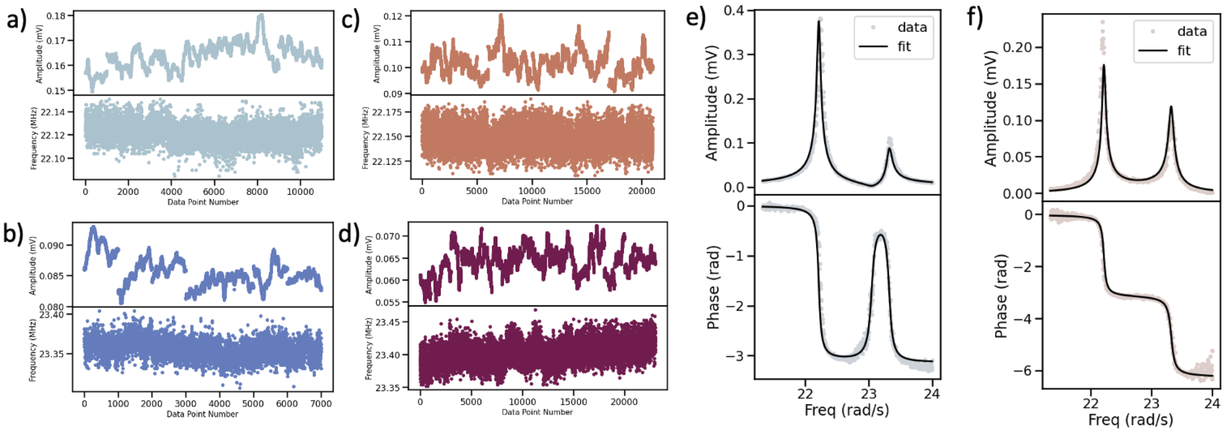

Figure S4: Additional figures for $N = 2$ cluster.
PLL measurements of amplitude and frequency plotted by data point number for corrected phase lock values of **a)** $\phi_1(\omega_a) = -0.47$ rad shown in light blue, **b)** $\phi_1(\omega_b) = -2.59$ rad shown in dark blue, **c)** $\phi_2(\omega_a) = -0.51$ rad shown in orange, **d)** $\phi_2(\omega_b) = -5.61$ rad shown in maroon. Least squares fit results shown as black solid lines over **e)** R1 amplitude (upper) and phase (lower) shown as gray data points and **f)** R2 amplitude (upper) and phase (lower) shown as light orange data points.

To observe the stability of the PLL measurements over time, we replot the data that is displayed as 2D boxplots in **Figure 3f-i**, as a function of data point number. The R1 PLL measurements are shown for phase lock values of $\phi_1(\omega_a)$, **Figure S4a**, and $\phi_1(\omega_b)$, **Figure S4b**. The R2 PLL measurements are shown for phase lock values of $\phi_2(\omega_a)$, **Figure S4c**, and $\phi_2(\omega_b)$, **Figure S4d**. With this representation, we see that the frequency is mostly stable for the $\phi_1(\omega_a)$, $\phi_1(\omega_b)$, and $\phi_2(\omega_a)$ phase locks. However, we do see an increasing trend for the $\phi_2(\omega_b)$ phase lock, which implies that these data are likely affected by the experimental phase lag during the PLL measurement and may account for error in the calculation of $\vec{p}$.

In the main text, we evaluate the accuracy of the $\vec{p}$ calculated from NETMAP by comparing each extracted value to expected values. To estimate the expected value of the coupling strength, we start with the equation(*23*)

$$\Delta\omega = \sqrt{\frac{(c/m_1)(c/m_2)}{\omega_1\omega_2}}$$

In this equation, $\omega_1 = \sqrt{\frac{k_1+c}{m_1}}$, $\omega_2 = \sqrt{\frac{k_2+c}{m_2}}$, and $\Delta\omega$ is approximated to be the difference between the antisymmetric mode frequency and the symmetric mode frequency, $\Delta\omega = \omega_A - \omega_S$. We simplify this equation by assuming the two resonators have equal mass, m, and equal intrinsic spring constants, k. We then normalize by the eigenfrequency of the uncoupled resonators, $\omega_0 = \sqrt{\frac{k}{m}}$.

$$\frac{\Delta\omega}{\omega_0} = c\sqrt{\frac{1}{k(k+c)}}$$

We can then solve for c, in which we define $\Omega = \frac{\Delta\omega}{\omega_0}$,

$$c = \frac{1}{2}\left(k\Omega^2 \pm \sqrt{(k\Omega^2)^2 + (2k\Omega)^2}\right)$$

We used the SciPy find peaks function in Python to estimate $\Delta\omega = 1.1$ MHz and $\omega_0 = \omega_S + \frac{\Delta\omega}{2} = 22.8$ MHz. Using these values and approximating $k = 1$ N/m, we obtain an estimate of $c = 0.05$ N/m.

To further validate NETMAP, we compare the NETMAP output parameters to those from Unity LS. The fit result of Unity LS, used for comparison in the main text, is shown as a solid black line overlayed on the experimental spectra, gray or light orange data points, in **Figure S4e,f**.

To highlight the sensitivity of LS to input guesses, we also performed LS with increasing our input guess of the two masses from $m_1 = m_2 = 10^{-17}$ kg to 10^{-16} kg. For this fit, we saw order of magnitude or more differences in the output values of k_2, c_1, m_2, b_2, and F that do not fall within the expected ranges discussed in the main text. While individually k_2 and m_2 are far from the expected values, the ratio of $\sqrt{\frac{k_2}{m_2}}$ gives an expected resonance frequency of ~16 MHz, within 7 MHz of the observed amplitude peaks. These correlated quantities, which are prevalent in our model, result in many possible solutions for least squares fitting, making this method reliant on the input guess solutions to find the residual minimum that is closest to the actual values. Combining NetMAP with LS fitting by inputting $\vec{p}$ as guess solutions (see SI for resulting $\vec{p}$ and R^2 values) may be an effective strategy to fit parameter values as close to the actual values as possible.

As a final comparison, we used LS to fit the vector weights of the two smallest singular values. In practice, we characterize the null-space dimension by the number of singular values λ such that $\lambda \ll 1$. For the $N = 1$ cluster, (**Figure 2**), we had only one singular value ($\lambda \sim 10^{-9}$), so the null-space was 1D. However, the $N = 2$ cluster (**Figure 3**) had three potential singular values ($\lambda_1 \sim 10^{-4}$, $\lambda_2 \sim 10^{-6}$, and $\lambda_3 \sim 10^{-8}$). We applied the α_i LS approach to the two smallest singular

values of the $N = 2$ system and found that all output values still fall within the expected ranges and the corresponding R^2 did not change significantly.

**Mechanical Parameter**	**LS with guess of $m_1 = m_2 = 10^{-16}$kg**	**α_i LS**
k_1 [N/m]	1	1
k_2 [N/m]	0.004	1.686
c_1 [N/m]	0.004	0.065
m_1 [10^{-17} kg]	5.022	5.247
m_2 [10^{-17} kg]	0.038	8.481
b_1 [10^{-11} kg/s]	1.452	2.250
b_2 [10^{-11} kg/s]	0.022	5.149
F [10^{-6} N]	0.112	1.790
R1 Amplitude R^2	−0.27	0.79
R1 Phase R^2	0.99	0.84
R2 Amplitude R^2	0.78	0.67
R2 Phase R^2	0.996	0.995

Table S1: LS with alternate guess solutions, LS to solve for vector weights of $N = 2$ cluster

Section S6: Additional Cluster Analysis ($N = 1$ and $N = 2$)

To further demonstrate NetMAP, we characterized additional clusters of size $N = 1$ and $N = 2$. We first characterized the $N = 1$ cluster by aligning the pump and probe over the resonator highlighted in **Figure S5a** and acquired amplitude and phase spectra. The resulting spectra had a single peak in the amplitude, gray data points in **Figure S5f upper**, and a corresponding phase that crossed through $\pi/2$ at resonance, gray data points in **Figure S5f lower**, consistent with a single, uncoupled resonator. To confirm the cluster size of $N = 1$, we took a SIM scan at $f = 17.64$ MHz, with the resulting amplitude spatial map shown in **Figure S5b** and phase in **Figure S5c**. In this scan we observed a single region of peak amplitude that had a constant phase, indicating that the cluster consisted of a single, uncoupled resonator. This region also corresponded in size and location to the driven resonator highlighted in **Figure S5a**.

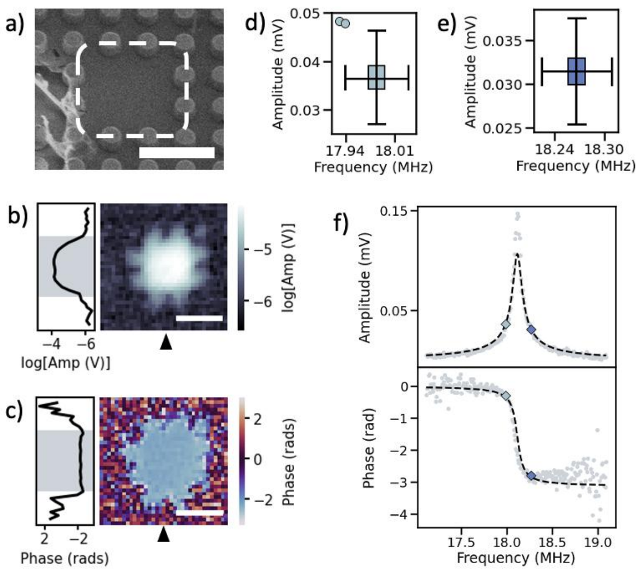

Figure S5: Additional $N = 1$ cluster.
a) SEM of driven uncoupled resonator, with pillar radii of 0.5 μm and pillar pitch 2 μm. Scale bar is 4 μm. **b)** Amplitude and **c)** phase spatial maps at a drive frequency of 17.64 MHz, scale bars are 5 μm. Phase spatial map shows uncorrected wrapped phase values. Black triangles on the bottom axis indicate the location of the vertical line scan on the left-side axis. 2D boxplot of PLL measurement distribution of frequency and amplitude for corrected phase lock values of **d)** $\phi_1(\omega_a) = -0.30$ rad and **e)** $\phi_2(\omega_b) = -2.79$ rad. 2D boxplots shows median, upper, and lower quartile ranges with whiskers that extend to include 1.5 IQR. Plotted circles represent datapoints that were outliers in both frequency and amplitude. **f)** Amplitude and corrected phase spectra of driven resonator. The diamond points in the amplitude spectrum (upper) correspond to the mean PLL measurements of amplitude ($\overline{A_1}(\omega_a) = 0.03684 \pm 0.00001$ mV and $\overline{A_1}(\omega_b) = 0.03146 \pm 0.00001$ mV) and frequency ($\overline{\Omega_1}(\omega_a)/2\pi = 17.98422 \pm 0.00005$ MHz and $\overline{\Omega_1}(\omega_b)/2\pi = 18.26637 \pm 0.00007$ MHz). The diamond points in the phase spectrum (lower) correspond to the locked phase values, $\phi_1(\omega_a)$ and $\phi_1(\omega_b)$, and the mean frequency values, $\overline{\Omega_1}(\omega_a)/2\pi$ and $\overline{\Omega_1}(\omega_b)/2\pi$. The black dotted line represents $|Z_1(\omega)|$ and $\phi_1(\omega)$ generated from the normalized $\vec{p}$.

We next measured $\omega_a, \omega_b, \vec{Z}(\omega_a)$ and $\vec{Z}(\omega_b)$, which are needed to compute $\boldsymbol{\mathcal{Z}}$. We chose target values of ω_a and ω_b to be on either side of the resonance peak, **Figure S5f upper.** We used the corrected phase spectra, **Figure S5f lower**, to map the chosen values of ω_a and ω_b to phase values for the PLL. The measured PLL time series of amplitude, $A(\omega_{a,b})$, and frequency, $\Omega(\omega_{a,b})/2\pi$, are shown as 2D boxplots in **Figure S5d,e**. Using the mean values of amplitude, $\overline{A}(\omega_a)$ and $\overline{A}(\omega_b)$, and frequency, $\overline{\Omega}(\omega_a)$ and $\overline{\Omega}(\omega_b)$, we obtain $\omega_a = \overline{\Omega}(\omega_a)$, $\omega_b = \overline{\Omega}(\omega_b)$, $\vec{Z}(\omega_a) = \{\overline{A}(\omega_a)e^{i\phi(\omega_a)}\}$, and $\vec{Z}(\omega_b) = \{\overline{A}(\omega_b)e^{i\phi(\omega_b)}\}$.

We then populated the matrix $\boldsymbol{\mathcal{Z}}$ with coefficients of the experimentally measured $\omega_a, \omega_b, \vec{Z}(\omega_a)$ and $\vec{Z}(\omega_b)$ and solved for the parameters vector $\vec{p}$. The resulting values are scaled to k and listed in Table S2. We found that each predicted parameter in $\vec{p}$ was consistent with the expected rang es discussed in the main paper. Moreover, by comparing the analytical $\vec{Z}_1(\omega)$ and $\vec{Z}_2(\omega)$ generated from $\vec{p}$ to experimental spectra, we found that the model can account for 95% of the variation in the data, with R^2 values listed in Table S2. We also compared the results

from NetMAP to those from Unity LS and found that m ($p = 0.35$), b ($p = 0.91$), and F ($p = 0.41$) all agree. We therefore conclude that NetMAP is proficient in characterizing the local cluster.

**Mechanical Parameter**	**NetMAP**	**Unity LS**
k [N/m]	1	1
m [10^{-17} kg]	7.718 ± 0.010	7.709
b [10^{-11} kg/s]	4.595 ± 2.814	4.293
F [10^{-7} N]	5.581 ± 0.527	6.017
Amplitude R^2	0.94	0.95
Phase R^2	0.97	0.97

Table S2: Results for additional $N = 1$ cluster from NetMAP and Unity LS

We characterized the additional $N = 2$ cluster by first aligning the pump and probe over the region highlighted as R1 in **Figure S6a** and taking amplitude and phase spectra. The amplitude spectrum, gray data points in **Figure S6j**, revealed two peaks that corresponded to phase changes that pass through $\sim\pi/2$ on resonance. To test whether these peaks correspond to the hybridized modes of a coupled pair, we took an SIM scan at $f = 15.45$ MHz, **Figure S6b,c**, and another at $f = 16.21$ MHz, **Figure S6d,e**. In the first scan, we saw two distinct high intensity area regions with nearly constant phase across each individual resonator. The first 6×6 µm region matched the size and location of the driven resonator, R1 highlighted in **Figure S6a**. The neighboring 6×6 µm region matched the size and location of the resonator to the right of R1, labelled as R2 in **Figure S6a**. Although we noticed smaller neighboring regions of amplitude maxima and constant phase, because we only observe two peaks in the amplitude spectra, we approximate other membrane motion to be due to weak coupling. We therefore approximate this system to be an $N = 2$ cluster.

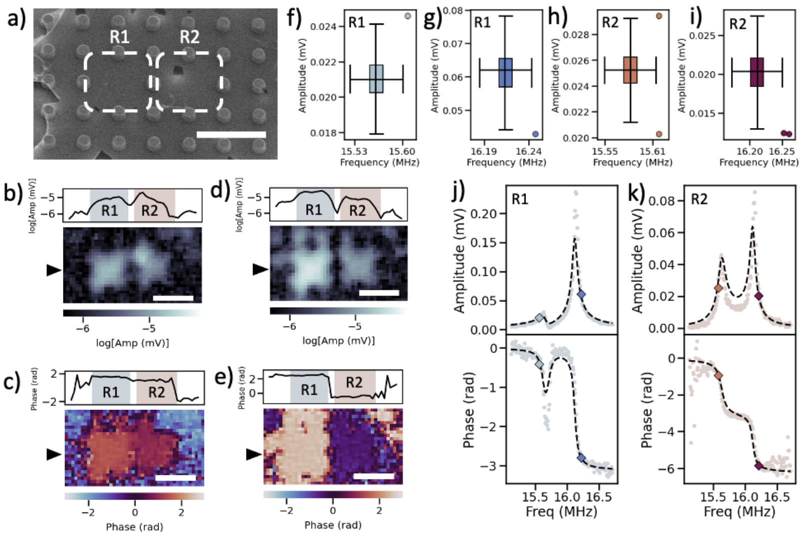

Figure S6: Additional $N = 2$ cluster.
a) SEM of driven resonator, R1, and neighboring coupled resonator, R2. Pillar radii are 0.5 μm and pillar pitch is 3 μm, scale bar is 6 μm. **b)** Amplitude and **c)** phase spatial maps taken at a drive frequency of $f_1 = 15.45$ MHz, scale bars are 5 μm. **d)** Amplitude and **e)** phase spatial maps taken at a drive frequency of $f_2 = 16.21$ MHz, scale bars are 5 μm. **c)** and **e)** phase spatial maps show uncorrected wrapped phase values. Black triangles on the left-side axes indicate the location of the vertical line scan on the left-side axis. 2D boxplots of PLL measurement distributions of frequency and amplitude for a phase lock of **f)** $\phi_1(\omega_a) = -0.41 \pm 0.15$ rad, **g)** $\phi_1(\omega_b) = -2.79 \pm 0.15$ rad, **h)** $\phi_2(\omega_a) = -0.93 \pm 0.81$ rad, and **i)** $\phi_2(\omega_b) = -5.85 \pm 0.81$. **j)** Amplitude (upper) and corrected phase (lower) of R1. Diamond points in the amplitude plot correspond to PLL measurements of amplitude ($\overline{A_1}(\omega_a) = 0.021010 \pm 0.000007$ mV and $\overline{A_1}(\omega_b) = 0.060903 \pm 0.000020$ mV) , and frequency ($\overline{\Omega_1}(\omega_a) = 15.56192 \pm 0.00009$ MHz and $\overline{\Omega_1}(\omega_b) = 16.21389 \pm 0.0004$ MHz). Diamond points in the phase plot correspond to the phase lock values, $\phi_1(\omega_a)$ and $\phi_1(\omega_b)$, and the mean frequency values, $\overline{\Omega_1}(\omega_a)$ and $\overline{\Omega_1}(\omega_b)$. The black dotted lines represent $|Z_1(\omega)|$ and $\phi_1(\omega)$ generated from the normalized $\vec{p}$. **k)** Amplitude (upper) and corrected phase (lower) of R2. Diamond points in the amplitude plot (upper) correspond to PLL measurements of amplitude ($\overline{A_2}(\omega_a) = 0.025206 \pm 0.000008$ mV and $\overline{A_2}(\omega_b) = 0.020166 \pm 0.000007$ mV) , and frequency ($\overline{\Omega_2}(\omega_a) = 15.58158 \pm 0.00007$ MHz and $\overline{\Omega_2}(\omega_b) = 16.21161 \pm 0.00004$ MHz). Diamond points in the phase plot (lower) correspond to the phase lock values, $\phi_2(\omega_a)$ and $\phi_2(\omega_b)$, and the mean frequency values, $\overline{\Omega_2}(\omega_a)$ and $\overline{\Omega_2}(\omega_b)$. The black dotted lines represent $|Z_2(\omega)|$ and $\phi_2(\omega)$ generated from the normalized $\vec{p}$.

To measure $\omega_a, \omega_b, \vec{Z}(\omega_a)$ and $\vec{Z}(\omega_b)$, we chose a target value of ω_a below the symmetric mode peak and ω_b above the antisymmetric mode peak, shown for R1 in **Figure S6j upper** and for R2 in **Figure S6k upper**. We used the corrected R1 phase spectra, **Figure S6j lower**, to map the chosen ω_a and ω_b to phase values of $\phi_1(\omega_a)$ and $\phi_1(\omega_b)$. Because the R2 phase spectrum off resonance was too noisy to accurately fit to a linear model, we corrected the R2 phase values with the R1 fit values of ϕ_0 and τ. With the probe positioned over R1, we acquired PLL time-series measurements for the two phase values, shown as 2D boxplots in **Figure S6f,g**. We repeated these PLL measurements for the second resonator by using the corrected R2 phase spectra, **Figure S6k lower**, to map ω_a and ω_b to phase values of $\phi_2(\omega_a)$ and $\phi_2(\omega_b)$. We positioned the probe over R2, fixed the pump over R1, and acquired PLL time-series measurements for each phase, shown

as 2D boxplots **Figure S6h,i**. We then used the PLL measurements to calculate $\omega_a = \left(\frac{1}{2}\right)(\overline{\Omega_1}(\omega_a) + \overline{\Omega_2}(\omega_a))$, $\vec{Z}(\omega_a) = \{\overline{A_1}(\omega_a)e^{i\phi_1(\omega_a)}, \overline{A_2}(\omega_a)e^{i\phi_2(\omega_a)}\}$, $\omega_b = \left(\frac{1}{2}\right)(\overline{\Omega_1}(\omega_b) + \overline{\Omega_2}(\omega_b))$, and $\vec{Z}(\omega_b) = \{\overline{A_1}(\omega_b)e^{i\phi_1(\omega_b)}, \overline{A_2}(\omega_b)e^{i\phi_2(\omega_b)}\}$.

Using the calculated values of ω_a, ω_b, $\vec{Z}(\omega_a)$, and $\vec{Z}(\omega_b)$, we populated the matrix $\boldsymbol{\mathcal{Z}}$ and solved for the parameters vector $\vec{p}$. We applied SVD to solve $\boldsymbol{\mathcal{Z}}\vec{p} = \vec{0}$ for the eight unknown components of $\vec{p}$, which are scaled by k_1 and listed in **Table S3**. We find that the values of k_2, c_1, m_1, m_2, b_1, and b_2 are all within the expected ranges discussed in the main text. Moreover, we observe that this set of parameters has predictive power over the experimentally measured spectral range, with R1 and R2 amplitude R^2 values ≥ 0.77 and the phase R^2 values ≥ 0.92 (See **Table S3**).

Mechanical Parameter	NetMAP	Unity LS	α_i LS
k_1 [N/m]	1 (scaling factor)	1	1
k_2 [N/m]	0.923 ± 0.299	0.664	1.301
c_1 [N/m]	0.021 ± 0.006	0.021	0.029
m_1 [10^{-17} kg]	10.035 ± 1.544	10.039	10.113
m_2 [10^{-17} kg]	9.695 ± 3.134	7.032	13.661
b_1 [10^{-11} kg/s]	4.336 ± 4.00	4.476	2.650
b_2 [10^{-11} kg/s]	7.396 ± 5.546	4.311	10.204
F [10^{-6} N]	0.883 ± 0.355	0.910	0.736
R1 Amplitude R^2	0.88	0.90	0.94
R1 Phase R^2	0.97	0.98	0.98
R2 Amplitude R^2	0.77	0.35	0.94
R2 Phase R^2	0.92	0.93	0.92

Table S3: Results for an additional $N = 2$ cluster

We also benchmarked NetMAP results against those form least squares fitting. In comparing the values from NetMAP to those of Unity LS, we find that all values agree within error for k_2 ($p = 0.38$), c_1($p = 0.99$), m_1 ($p = 0.998$), m_2 ($p = 0.40$), b_1 ($p = 0.97$), b_2 ($p = 0.58$), and F ($p = 0.94$). However, the predicted power of the Unity LS is poor for the R2 amplitude, as the model only accounts for 35% of variation in the experimental data (See **Table S3**). However, the method that had the best predictive power over the tested experimental range, was when we used LS to fit the vector weights corresponding to the two smallest singular values, with R1 and R2 amplitude and phase R^2 values all ≥ 0.92.

REFERENCES AND NOTES

1. J. Zhang, G. Pagano, P. W. Hess, A. Kyprianidis, P. Becker, H. Kaplan, A. V. Gorshkov, Z. X. Gong, C. Monroe, Observation of a many-body dynamical phase transition with a 53-qubit quantum simulator. *Nature* **551**, 601–604 (2017).

2. F. Arute, K. Arya, R. Babbush, D. Bacon, J. C. Bardin, R. Barends, R. Biswas, S. Boixo, F. G. S. L. Brandao, D. A. Buell, B. Burkett, Y. Chen, Z. Chen, B. Chiaro, R. Collins, W. Courtney, A. Dunsworth, E. Farhi, B. Foxen, A. Fowler, C. Gidney, M. Giustina, R. Graff, K. Guerin, S. Habegger, M. P. Harrigan, M. J. Hartmann, A. Ho, M. Hoffmann, T. Huang, T. S. Humble, S. V. Isakov, E. Jeffrey, Z. Jiang, D. Kafri, K. Kechedzhi, J. Kelly, P. V. Klimov, S. Knysh, A. Korotkov, F. Kostritsa, D. Landhuis, M. Lindmark, E. Lucero, D. Lyakh, S. Mandrà, J. R. McClean, M. McEwen, A. Megrant, X. Mi, K. Michielsen, M. Mohseni, J. Mutus, O. Naaman, M. Neeley, C. Neill, M. Y. Niu, E. Ostby, A. Petukhov, J. C. Platt, C. Quintana, E. G. Rieffel, P. Roushan, N. C. Rubin, D. Sank, K. J. Satzinger, V. Smelyanskiy, K. J. Sung, M. D. Trevithick, A. Vainsencher, B. Villalonga, T. White, Z. J. Yao, P. Yeh, A. Zalcman, H. Neven, J. M. Martinis, Quantum supremacy using a programmable superconducting processor. *Nature* **574**, 505–510 (2019).

3. I. Bloch, Ultracold quantum gases in optical lattices. *Nat. Phys.* **1**, 23–30 (2005).

4. P. A. Deymier, *Acoustic Metamaterials and Phononic Crystals* (Springer-Verlag, ed. 1, 2013).

5. J. D. Joannopoulos, S. G. Johnson, J. N. Winn, R. D. Meade, *Photonic Crystals: Molding the Flow of Light* (Princeton Univ. Press, ed. 2, 2008).

6. J. J. Hopfield, Neural networks and physical systems with emergent collective computational abilities. *Proc. Natl. Acad. Sci. U.S.A.* **79**, 2554–2558 (1982).

7. C. Mead, Neuromorphic electronic systems. *Proc. IEEE* **78**, 1629–1636 (1990).

8. X. C. Tong, *Functional Metamaterials and Metadevices* (Springer Nature, 2018).

9. O. R. Bilal, A. Foehr, C. Daraio, Reprogrammable phononic metasurfaces. *Adv. Mater.* **29**, 91125 (2017).

10. J. Cha, C. Daraio, Electrical tuning of elastic wave propagation in nanomechanical lattices at MHz frequencies. *Nat. Nanotechnol.* **13**, 1016–1020 (2018).

11. D. Hatanaka, A. Bachtold, H. Yamaguchi, Electrostatically induced phononic crystal. *Phys. Rev. Appl.* **11**, 1 (2019).

12. J. Cha, K. W. Kim, C. Daraio, Experimental realization of on-chip topological nanoelectromechanical metamaterials. *Nature* **564**, 229–233 (2018).

13. O. Florez, G. Arregui, M. Albrechtsen, R. C. Ng, J. Gomis-Bresco, S. Stobbe, C. M. Sotomayor-Torres, P. D. García, Engineering nanoscale hypersonic phonon transport. *Nat. Nanotechnol.* **17**, 947–951 (2022).

14. G. Csaba, W. Porod, Coupled oscillators for computing: A review and perspective. *Appl. Phys. Rev.* **7**, 011302 (2020).

15. M. H. Matheny, J. Emenheiser, W. Fon, A. Chapman, A. Salova, M. Rohden, J. Li, M. Hudoba de Badyn, M. Posfai, L. Duenas-Osorio, M. Mesbahi, J. P. Crutchfield, M. C. Cross, R. M. D. Souza, M. L. Roukes, Exotic states in a simple network of nanoelectromechanical oscillators. *Science* **363**, 1057 (2019).

16. D. Miller, A. Blaikie, B. J. Alemán, Nonvolatile rewritable frequency tuning of a nanoelectromechanical resonator using photoinduced doping. *Nano Lett.* **20**, 2378–2386 (2020).

17. G. Luo, Z. Z. Zhang, G. W. Deng, H. O. Li, G. Cao, M. Xiao, G. C. Guo, L. Tian, G. P. Guo, Strong indirect coupling between graphene-based mechanical resonators via a phonon cavity. *Nat. Commun.* **9**, 3–8 (2018).

18. H. Okamoto, A. Gourgout, C. Y. Chang, K. Onomitsu, I. Mahboob, E. Y. Chang, H. Yamaguchi, Coherent phonon manipulation in coupled mechanical resonators. *Nat. Phys.* **9**, 480–484 (2013).

19. J. Doster, S. Hoenl, H. Lorenz, P. Paulitschke, E. M. Weig, Collective dynamics of strain-coupled nanomechanical pillar resonators. *Nat. Commun.* **10**, 5246 (2019).

20. H. Motulsky, A. Ransnas, Fitting curves to data using nonlinear regression: A practical and nonmathematical review. *J. Fed. Am. Soc. Exp. Biol.* **1**, 365–374 (1987).

21. A. T. Winfree, Biological rhythms and the behavior of populations of coupled oscillators. *J. Theor. Biol.* **16**, 15–42 (1967).

22. P. Ashwin, O. Burylko, Weak chimeras in minimal networks of coupled phase oscillators. *Chaos* **25**, 013106 (2015).

23. L. Novotny, Strong coupling, energy splitting, and level crossings: A classical perspective. *Am. J. Phys.* **78**, 1199–1202 (2010).

24. D. Miller, B. Alemán, Spatially resolved optical excitation of mechanical modes in graphene NEMS. *Appl. Phys. Lett.* **115** (2019).

25. D. Davidovikj, J. J. Slim, S. J. Cartamil-Bueno, H. S. J. Van Der Zant, P. G. Steeneken, W. J. Venstra, Visualizing the motion of graphene nanodrums. *Nano Lett.* **16**, 2768–2773 (2016).

26. R. De Alba, F. Massel, I. R. Storch, T. S. Abhilash, A. Hui, P. L. McEuen, H. G. Craighead, J. M. Parpia, Tunable phonon-cavity coupling in graphene membranes. *Nat. Nanotechnol.* **11**, 741–746 (2016).

27. B. Carter, U. F. Hernandez, D. J. Miller, A. Blaikie, V. R. Horowitz, B. J. Alemán, Coupled nanomechanical graphene resonators: A promising platform for scalable NEMS networks. *Micromachines* **14**, 2103 (2023).

28. A. Blaikie, D. Miller, B. J. Alemán, A fast and sensitive room-temperature graphene nanomechanical bolometer. *Nat. Commun.* **10**, 4726 (2019).

29. V. R. Horowitz, B. Carter, U. F. Hernandez, T. Scheuing, B. J. Alemán, Validating an algebraic approach to characterizing resonator networks. *Sci. Rep.* **14**, 1325 (2024).

30. I. W. Frank, D. M. Tanenbaum, A. M. van der Zande, P. L. McEuen, Mechanical properties of suspended graphene sheets. *J. Vac. Sci. Technol. B Microelectron. Nanometer Struct.* **25**, 2558–2561 (2007).

31. R. de Alba, I. R. Storch, J. M. Parpia, H. G. Craighead, T. S. Abhilash, A. Hui, Temperature-dependence of stress and elasticity in wet-transferred graphene membranes. *J. Appl. Phys.* **123**, 095109 (2018).

32. R. A. Barton, B. Ilic, A. M. van der Zande, W. S. Whitney, P. L. McEuen, J. M. Parpia, H. G. Craighead, High, size-dependent quality factor in an array of graphene mechanical resonators. *Nano Lett.* **11**, 1232–1236 (2011).

33. T. J. Booth, P. Blake, R. R. Nair, D. Jiang, E. W. Hill, U. Bangert, A. Bleloch, M. Gass, K. S. Novoselov, M. I. Katsnelson, A. K. Geim, Macroscopic graphene membranes and their extraordinary stiffness. *Nano Lett.* **8**, 2442–6 (2008).

34. Y. Wang, J. Lee, X.-Q. Zheng, Y. Xie, P. X.-L. Feng, Hexagonal Boron nitride phononic crystal waveguides. *ACS Photonics* **6**, 3225–3232 (2019).

35. M. H. J. De Jong, M. A. Ten Wolde, A. Cupertino, S. Gröblacher, P. G. Steeneken, R. A. Norte, Mechanical dissipation by substrate-mode coupling in SiN resonators. *Appl. Phys. Lett.* **121**, 032201 (2022).

36. M. Šiškins, E. Sokolovskaya, M. Lee, S. Mañas-Valero, D. Davidovikj, H. S. J. van der Zant, P. G. Steeneken, Tunable strong coupling of mechanical resonance between spatially separated $FePS_3$ nanodrums. *Nano Lett.* **22**, 36–42 (2022).

37. D. Hatanaka, I. Mahboob, K. Onomitsu, H. Yamaguchi, Phonon waveguides for electromechanical circuits. *Nat. Nanotechnol.* **9**, 520–524 (2014).

38. L. N. Trefethen, D. Bau III, *Numerical Linear Algebra* (Society for Industrial and Applied Mathematics, 1997).

39. J. Doster, T. Shah, T. Fösel, P. Paulitschke, F. Marquardt, E. M. Weig, Observing polarization patterns in the collective motion of nanomechanical arrays. *Nat. Commun.* **13**, 1–7 (2022).

40. D. Vodenicarevic, N. Locatelli, F. Abreu Araujo, J. Grollier, D. Querlioz, A nanotechnology-ready computing scheme based on a weakly coupled oscillator network. *Sci. Rep.* **7**, 44772 (2017).

41. T. Faust, J. Rieger, M. J. Seitner, J. P. Kotthaus, E. M. Weig, Coherent control of a classical nanomechanical two-level system. *Nat. Phys.* **9**, 485–488 (2013).

42. A. Kumar, P. Mohanty, Autoassociative memory and pattern recognition in micromechanical oscillator network. *Sci. Rep.* **7**, 1–9 (2017).

43. J. Lee, M. D. LaHaye, P. X. L. Feng, Design of strongly nonlinear graphene nanoelectromechanical systems in quantum regime. *Appl. Phys. Lett.* **120**, 014001 (2022).

44. A. Reserbat-Plantey, D. Kalita, Z. Han, L. Ferlazzo, S. Autier-Laurent, K. Komatsu, C. Li, R. Weil, A. Ralko, L. Marty, S. Guéron, N. Bendiab, H. Bouchiat, V. Bouchiat, Strain superlattices and macroscale suspension of graphene induced by corrugated substrates. *Nano Lett.* **14**, 5044–5051 (2014).

45. J. R. Taylor, *An Introduction to Error Analysis* (University Science Books, ed. 2, 1997).
